# Species-level detection of thrips and whiteflies on yellow sticky traps using YOLO-based deep learning detection models

**DOI:** 10.3389/fpls.2025.1668795

**Published:** 2025-11-18

**Authors:** Broes Laekeman, Jochem Bonte, Wannes Dermauw, Annelies Christiaens, Bruno Gobin, Johan Van Huylenbroeck, Emmy Dhooghe, Peter Lootens

**Affiliations:** 1 Plant Sciences Unit, Flanders Research Institute for Agriculture, Fisheries and Food (ILVO), Merelbeke-Melle, Belgium; 2 Department of Plants and Crops, Faculty of Bioscience Engineering, Ghent University (UGent), Gent, Belgium; 3 Viaverda, Destelbergen, Belgium

**Keywords:** automated pest monitoring, integrated pest management (IPM), artificial intelligence (AI), smart traps, *Frankliniella occidentalis*, *Echinothrips americanus*, *Bemisia tabaci*, *Trialeurodes vaporariorum*

## Abstract

As of today, pest insects such as thrips and whiteflies cause the loss of 20% - 40% of the global agricultural yield. To reduce chemical pesticide use while maintaining high-quality horticultural standards, early detection of pest infestations is essential. Although AI-assisted pest monitoring systems using sticky trap images exist today, none currently enable effective species-level detection of thrips and/or whiteflies. However, early species-level identification would allow for more targeted, species-specific control strategies, leading to reduced, localized, and more efficient pesticide application. Therefore, in this study, we evaluated the potential and limitations of real-time species-level detection of thrips (*Frankliniella occidentalis* and *Echinothrips americanus*) and whiteflies (*Bemisia tabaci* and *Trialeurodes vaporariorum*) using non-microscopic, RGB yellow sticky trap images and recent YOLO-based deep learning detection models. To this end, a balanced and labelled image dataset was gathered, consisting of the studied pest species, caught on one type of yellow sticky trap. Subsequently, various versions of the YOLO11 and YOLO-NAS detection model architectures were trained and tested using this dataset at various (digitally reduced) pixel resolutions. All tested high-resolution dataset (pixel size: 5 µm) models achieved species-level detection of the studied pests on an independent test dataset (mAP@50: 79% - 89% | F1@50: 74% - 87%). Even the smallest model (YOLO11n) delivered feasible macro-averaged (mAP@50: 80% | F1@50: 77%) and classwise performance scores (AP@50: 72% - 85% | F1@50: 68% - 82%). The minimum required pixel resolution for feasible species-level detection in greenhouse horticulture was identified as 80 *µm* for both the YOLO11n and YOLO11x models, enabling the use of modern smartphones, action cameras, or low-cost standalone camera modules. Combined with the low complexity and decent performance of the YOLO11n model, these results demonstrate the potential of feasible, real-time, automated species-level monitoring of (yellow) sticky traps in greenhouse horticulture. Future research should focus on extending this technology to additional pest species, sticky trap types, and ambient light conditions.

## 1 Introduction

According to the United Nations (2022), the global human population will reach 9.7 billion by 2050, requiring an increase in food production of approximately 30% - 60% compared to the baseline period of 2005-2010 (Food and Agriculture Organization of the United Nations, 2024; van Dijk et al., 2021). Considering the annual global crop loss of 20% - 40% due to pest insects today (Food and Agriculture Organization of the United Nations, 2024; Gula, 2023), combined with the increasing pressure on the use and authorization of chemical pesticides, this will be a significant challenge. The switch to a more preventive, efficient and integrated pest management (IPM) strategy will therefore be key, requiring fast and objective detection combined with effective local pest control techniques.

Among all flying insects, the Thysanoptera order (better known as thrips) and Aleyrodidae family (better known as whiteflies) are widely distributed across the globe in both open field agriculture and greenhouse horticulture (Fiallo-Olivé et al., 2019; Mound, 2009; Perring et al., 2018). However, only a small fraction (< 1%) of these thrips and whitefly species are recognized as major agricultural pests. Thrips pests cause damage by feeding on leaf, flower and fruit tissues, which diminishes plant vigour and aesthetic quality. Additionally, some can act as vectors of harmful plant viruses, such as tospoviruses (Mound and Teulon, 1995; Mound et al., 2022). Whitefly pests, by contrast, cause damage by feeding on plant phloem and by excreting honeydew on the leaves, promoting fungal growth (e.g. sooty mould and powdery mildew) (Fiallo-Olivé et al., 2019; Navas-Castillo et al., 2011). Furthermore, some whitefly pests are well-known as vectors of various plant viruses, including begomoviruses, criniviruses, ipomoviruses, torradoviruses and some carlaviruses (Fiallo-Olivé et al., 2019; Navas-Castillo et al., 2011).

As both insect types are relatively small (thrips: 0.5–2 mm | whiteflies: 1–3 mm) and generally occur on the abaxial leaf side, they are easily overlooked by growers (Manners and Duff, 2017; Navas-Castillo et al., 2011). Combined with the high fecundity, short generation times and favourable climate inside horticultural greenhouses, this often results in exponential pest development (Manners and Duff, 2017; Perring et al., 2018). As of today, the western flower thrips (*Frankliniella occidentalis*), silverleaf whitefly (*Bemisia tabaci*) and to a lesser extent the greenhouse whitefly (*Trialeurodes vaporariorum*) are considered among the most problematic agricultural pests due to their global spread, polyphagous nature and - most importantly - their virus-spreading behaviour (Fiallo-Olivé et al., 2019; Kanakala and Ghanim, 2019; Navas-Castillo et al., 2011). Another widely distributed pest species across greenhouses is the so-called poinsettia thrips (*Echinothrips americanus*). Although it is not recognized as a plant virus vector, *E. americanus* is also considered an important horticultural pest due to its polyphagous nature, limited initial plant damage and rather low mobility, which increases the risk for delayed detection and exponential growth (Pijnakker et al., 2018; Pundt, 2024; Vierbergen et al., 2006).

Today, monitoring for these pests is generally carried out using glue-covered, brightly coloured (chromotropic) paper/plastic cards (also referred to as *sticky traps*), followed by frequent (e.g. daily/weekly) manual inspection. Subsequently, all present insects are manually identified using the key morphological traits. *E. americanus* adults are dark brown with red bands between the abdominal segments and have a unique white patch at the base of their dark wings (Mound et al., 2025). By contrast, *F. occidentalis* adults are smaller, slender, and vary in colour from pale yellow to nearly black, with narrow, fringed wings and no distinct white markings on the wings (Mound et al., 2025). In *T. vaporariorum*, the anterior margin of the forewing is curved, while in *B. tabaci* it is straight (European and Mediterranean Plant Protection Organization, 2004). Furthermore, in resting position, the wings of *B. tabaci* look more narrow and are pointed posteriorly compared to *T. vaporariorum* (European and Mediterranean Plant Protection Organization, 2004). Lastly, *B. tabaci* adults are generally somewhat smaller and have a darker yellow body compared to *T. vaporariorum* adults (European and Mediterranean Plant Protection Organization, 2004).

Due to its high attractivity to a wide range of insects, the yellow sticky trap (YST) is mostly used for monitoring. Although the material cost is fairly limited, the human labour cost and the non-continuous nature of this method still leave room for improvement. In addition, most personnel are not equipped or trained for accurate pest identification, particularly at the species level. However, continuous species-level monitoring of harmful pests would allow for timely, local and species-specific (non)chemical countermeasures, while taking into account the biology and phenology of the targeted species. Consequently, both the efficiency and efficacy of chemical pesticides would increase, while reducing the dosage, cost, environmental impact and risk of pesticide resistance. Therefore, this approach perfectly aligns with the European Union IPM strategies (The European Parliament and the Council of the European Union, 2009). Furthermore, more reliable risk assessments of virus transmission and the associated economic impact could be made using species-specific detections. Lastly, the success of the applied pest management strategy could also be quantified using such a system.

In an attempt to automate and objectively standardise the monitoring process of pests on sticky traps, various solutions combining a sticky trap with an optical sensor, have been proposed in the literature over the last three decades. A detailed overview can be found in the review articles by Lima et al. (2020), Preti et al. (2021), and Teixeira et al. (2023). Originally, this development started as static, automated and non-specific insect counting systems using basic optical sensors. Later, basic image processing techniques (e.g. filters, binarisation, colour space transformations, thresholding, etc.) were introduced and further evolved into mobile pest differentiation systems using machine learning techniques (e.g. k-means clustering, support vector machines, etc.) (Lima et al., 2020; Preti et al., 2021; Teixeira et al., 2023). During the last decade, many general (e.g. RetinaNet, Faster R-CNN, YOLO) and custom made/adapted versions of general deep learning (DL) model architectures [e.g. PestNet by Liu et al. (2019), TPest-RCNN by Li et al. (2021)] have been reported in the literature. These models allow for more specific pest detection on sticky traps, with generally good performance (mean average precision (mAP), calculated at an intersection over union (IoU) threshold of 50% - mAP@50: approx. 70% - 95%) (Teixeira et al., 2023).

When focusing on recent (2020 - present), good performing (mAP@50: ≥ 80%) DL-based sticky trap detection systems in the literature that specifically target thrips and/or whiteflies, the reported models are mainly (adapted) versions of the YOLO (Niyigena et al., 2023; Teixeira et al., 2023; Wang et al., 2021, 2024; Zhang et al., 2023) and Faster R-CNN (Li et al., 2021; Niyigena et al., 2023; Teixeira et al., 2023; Wu et al., 2024) model architectures. Despite the inclusion of both pest types in some studies, surprisingly only few articles address species-level determination of these pests. While some researchers specify the exact thrips and whitefly species studied, they either exclude other species from the same order/family (Espinoza et al., 2016; Sun et al., 2017; Xia et al., 2015) or group related species under a single detection class (Bauch and Rath, 2005), therefore avoiding the need for species-level differentiation.

To the best of the authors’ knowledge, only Niyigena et al. (2023) have currently reported a detection model capable of differentiating *Scirtothrips dorsalis* from other thrips species (grouped as a single class) using high-quality YST smartphone images (pixel resolution: 17 *µm*). Regarding whitefly species differentiation, only Böckmann et al. (2021) described a differentiation model for *B. tabaci* and *T. vaporariorum* on RGB sticky trap images, using a bag of visual words approach. However, its success was rather limited (*B. tabaci*: recall = 72% and precision = 26% | *T. vaporariorum*: recall = 54% and precision = 98%). In contrast, Gutierrez et al. (2019) reported detection models (SDD and Faster R-CNN) for *B. tabaci* and *T. vaporariorum* adults and eggs, but performance for adult detection remained rather modest (precision: 27% - 74%). However, note that the latter models were trained on close-up images of pests/eggs on plants, not on sticky trap images. Finally, as a general remark, most studies only report performance metrics for the originally obtained test dataset, limiting a critical assessment of the models’ generalization properties.

Therefore, in this research, we propose a proof-of-concept species-level detection system for two of the currently most occurring/damaging thrips (*F. occidentalis* and *E. americanus*) and whitefly (*B. tabaci* and *T. vaporariorum*) species in the Belgian and Dutch greenhouse horticultural sector, using non-microscopic, RGB yellow sticky trap images and recent DL models. To enable real-time detection, two state-of-the-art one-step DL detector architectures were selected, being the recently developed, relatively fast and good performing YOLO11 (Jocher et al., 2025) and YOLO-NAS (Aharon et al., 2024) model families. As a first step, the potential of DL-based species-level detection on sticky trap images was explored. This was done by training various versions, diverging in complexity, of the selected model architectures on a dedicated, high-resolution dataset. Next, the minimum required pixel resolution for feasible species-level thrips and whitefly detection in greenhouse horticulture was determined. This was achieved by training a selection of the proposed models on digitally transformed reduced-resolution datasets. During both steps, the influence of the model architecture and model size on the performance were studied. Furthermore, also the model generalization was studied using both an internal (subset of the original dataset) and external (additional independent dataset) test dataset. As a last step, the obtained theoretical minimum required pixel resolution was translated to various potential (low-cost) sticky trap image acquisition setups. To our knowledge, this is the first study enabling species-level detection of both thrips and whiteflies using RGB sticky trap images and YOLO-based detection models.

## 2 Materials and methods

### 2.1 Dataset acquisition

#### 2.1.1 Pest insect rearing and sticky trap collection

To obtain a heterogeneous, high-quality collection of insect-covered yellow sticky traps (YSTs) of various insect densities, residence times and age, *F. occidentalis* (thrips), *T. vaporariorum* (whitefly) and *B. tabaci* (whitefly) strains were reared inside insect-proof cages (Vermandel, The Netherlands) in physically isolated greenhouses/growing chambers at ILVO (Merelbeke-Melle, Belgium). The *F. occidentalis* strain, previously described by De Rouck et al. (2024), was reared on bean pods (*Phaseolus vulgaris*) with addition of pollen (Nutrimite, Biobest, Belgium) inside passively ventilated plastic containers. The *T. vaporariorum* strain was collected from a natural infection in an ILVO greenhouse and was reared on cucumber plants (*Cucumis sativus*). Both strains were reared in separate cages within the same ILVO greenhouse with an indoor temperature of 20.1 ± 1.7 °C and 55.0 ± 10.9% relative humidity (RH). The *B. tabaci* strain (MED biotype), previously described by Mocchetti et al. (2025), was reared on tobacco plants (*Nicotiana tabacum*) inside a separate ILVO growing chamber at 23.5 ± 1.1 °C and 60 ± 3% RH.

Over the course of several months, individuals of all insect populations were regularly caught on one commonly used type of (wet glue) YST in Belgium (Horiver Wetstick, Koppert België B.V., Belgium). This was done by hanging the YSTs inside the rearing cages/containers for several days. Meanwhile, the same type of YST containing a mix of *E. americanus* (thrips) and other insect species (other than thrips or whiteflies) was collected for two weeks in the greenhouses of Viaverda (Destelbergen, Belgium) after a natural infestation of pot plants. The indoor temperature and RH inside the greenhouses were, respectively, 24.9 ± 5.3 °C and 55.7 ± 16.5%. All sticky traps were stored, protected from ambient light, inside opaque plastic containers until image acquisition (several days to approximately one year) and were later used to construct the so-called *internal dataset*.

Lastly, to study the generalization of the models, also a smaller, independent additional collection of four YSTs was obtained, containing a mixture of all studied pest species and other non-thrips/-whitefly insects. This collection was acquired at the end of the insect rearing by moving a previously collected *E. americanus* (thrips) YST across all pest cultivations. Depending on the size and vigour of the pest populations, the YST was left inside each location for several hours to several days until at least ten individuals per pest species were caught on each sticky trap. The mixed YSTs were analogously stored inside opaque plastic containers until image acquisition (several days to months) and were later used to construct the so-called *external dataset*.

#### 2.1.2 Sticky trap image acquisition

**Figure 1:**
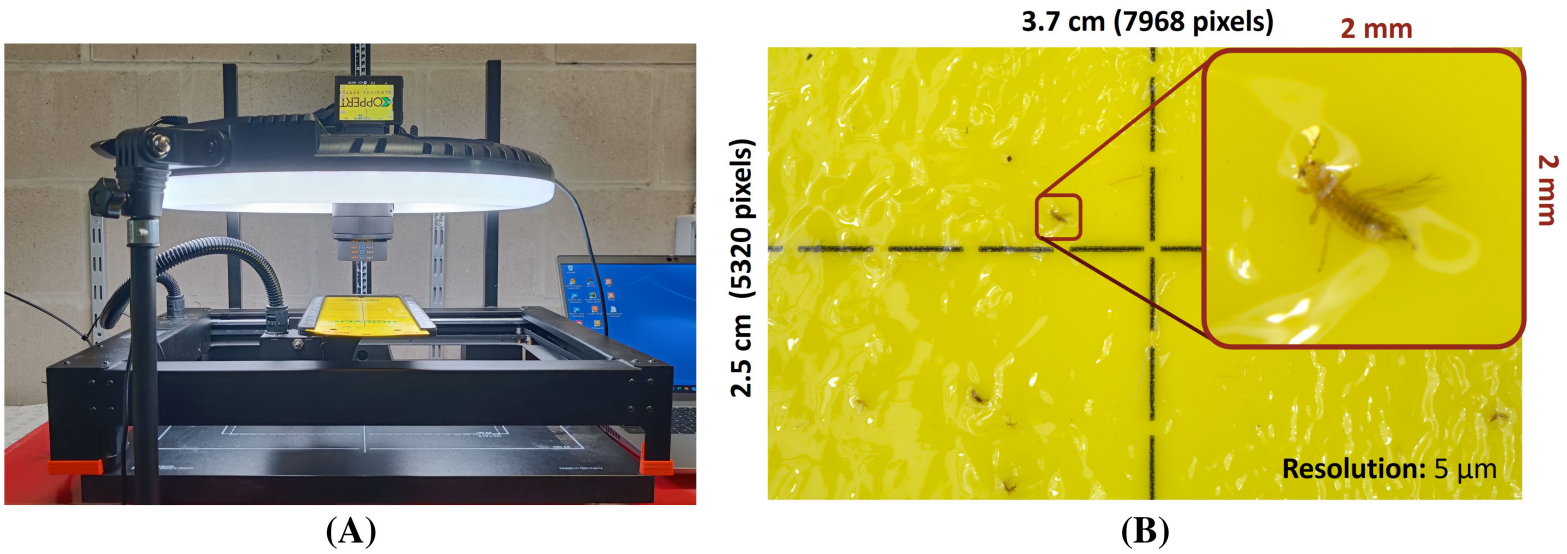
**(A)** Overview of the automated, high-resolution (pixel size: 5 *µm*) image acquisition setup and **(B)** a zoomed example image for *F*. *occidentalis*.

In order to study the potential of species-level pest detection using non-microscopic, high-resolution RGB images, all sticky traps were photographed using a standardized and automated image acquisition setup (see **Figure 1A**). This setup consisted of a 42.4 MP high-resolution DSLR camera (Sony *α*7R III, Sony Group Corporation, Japan) with macro lens (Sony FE 50mm F2.8 macro, Sony Group Corporation, Japan), a repro photography stand (Hama, Germany), a circular LED light (LED Ringlamp LR-480, StudioKing, The Netherlands) and a motorized xy gantry (XPlotter, PineconeRobotics, China) with a 3D printed sticky trap mount. The LED light was adjusted to 5500 K (built-in driver) and 2170 Lux (Testo 545 digital Lux meter, Testo AG, Germany), measured in the center of the YST. The camera height was adjusted to the minimum focus distance of the lens (16 cm) after which it was focussed on a paper black/white block pattern. This resulted in a field of view of roughly 3.7 cm x 2.5 cm (7968 x 5320 pixels) per image with a pixel size of roughly 5 *µm* (see **Figure 1B**).

All images were taken in manual mode (aperture: f/11 | shutter speed: 1” | ISO: 100) using the corresponding Imaging Edge Desktop - Remote software (v1.2.00.02130 | Sony Group Corporation, Japan) and saved in RAW format (*.ARW). The YST was automatically moved between images by the xy gantry, resulting in 30 images per sticky trap without image overlap. This was needed to avoid any potential data leakage between the training, validation and test datasets. Both sides of the YSTs (A: side with printed grid | B: non-printed side) were photographed using the same protocol. Image acquisition was spread over multiple days and grey card (Control-card, Novoflex, Germany) images for white balance correction were taken at the start and end of each acquisition day.

#### 2.1.3 Image processing and dataset labelling

##### 2.1.3.1 High-resolution datasets

All original images (*.ARW format) were corrected (white balance and lens correction) using the open source Darktable software (v4.4.1) and saved as 8-bit *.jpg images. Subsequently, all images were cropped to a central region of interest of 90% of the original image size to fully exclude any potential image overlap and data-leakage during training/testing. Next, bounding box labels (four classes: one for each studied thrips/whitefly species) were generated using a dedicated Python script based on colour space conversion and image thresholding or an early version of the trained detection model in a later phase. All bounding box labels were later manually verified using the free browser version of CVAT (v2.30.0 | CVAT.ai Corporation, Palo Alto, CA, USA) after which the images were split into smaller image patches, matching the neural network’s image input dimensions (640 x 640 pixels | 3.2 x 3.2 mm). Other insects (mainly originating from the *E. americanus* greenhouse), not belonging to any of the four studied pest species were left unlabelled in the dataset. The described process is visualized in **Supplementary Figure S1** in the **Supplementary Material File**.

**Figure 2:**
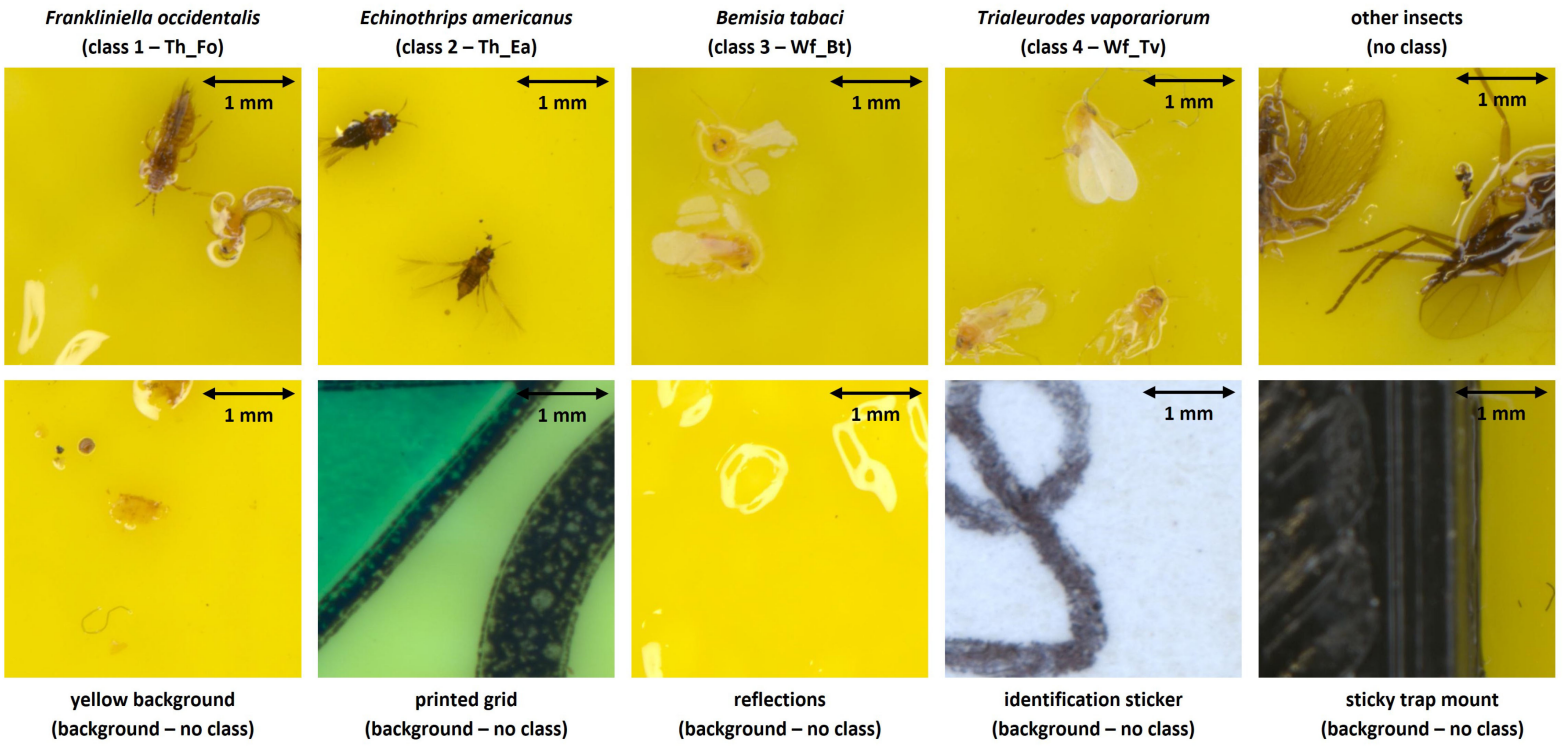
Overview of the defined image patch types in both the internal and external high-resolution (pixel size: 5 *µm*) image datasets. The dimensions of each image patch were 640 x 640 pixels, corresponding to 3.2 x 3.2 mm.

Next, all useful pest image patches were separated from the background (without insect labels) and blurry/dubious image patches using CVAT. Background patches (BG) were subsequently further divided into five subclasses (yellow background, printed grid, light reflections, identification sticker and sticky trap mount) per original pest species dataset. In order to prevent misclassification of other, non-studied insects, an additional image patch dataset (without labels) of all other present insects was also gathered (see **Figure 2**).

The high-resolution dataset was subsequently obtained by homogeneous sampling of 1,000 labels (+ the corresponding image patches) of each studied pest class, 1,000 patches of non-studied insects and 20% BG patches, spread over each background type according to its relevance (25% yellow background | 25% printed grid | 25% reflections | 10% identification sticker | 15% sticky trap mount). Finally, this dataset was randomly split into the training (60%), validation (20%) and test (20%) subsets and is further referenced to in this document as the *internal dataset*.

The additional collection of mixed insects YST images was processed analogously, but now only a test dataset was gathered consisting of 54 homogeneously sampled insect labels (+ the corresponding image patches) per studied class (maximum available balanced dataset size), combined with 54 image patches of other non-studied insects (mainly originating from the *E. americanus* greenhouse) and 20% BG patches. This dataset is further referenced to in this research as the *external dataset*.

##### 2.1.3.2 Reduced-resolution datasets

In order to study the effect of the image (pixel) resolution on the model’s performance/generalization, both the internal and external high-resolution datasets were digitally transformed into reduced-resolution datasets (theoretical pixel size - TPS: 10 *µm*, 20 *µm*, 40 *µm*, 80 *µm*, 160 *µm*, 320 *µm* and 640 *µm* | see **Supplementary Figure S2** in the **Supplementary Material File**). This was done by resizing the original image patch (640 x 640 pixels) to a smaller dimension (factor 1*/*2*n* | *n* ∈ N) using bilinear interpolation, followed by resizing it back to the original image patch dimension, also using bilinear interpolation. This way, the original dimension (640 x 640 pixels) and field of view (3.2 x 3.2 mm) were maintained in all image patches, while the included pixel information/detail originated from a (theoretical) lower original image resolution (= larger TPS). In order to avoid any potential influence of the resolution downscaling process, a dataset of the original resolution (TPS: 5 *µm*) was also obtained using the same method.

##### 2.1.3.3 Python environment

All image and dataset processing steps were performed in Python (v3.8.19) using the following main libraries: plantcv (v4.3.1), opencv-python (v4.10.0.82) and pillow (v10.2.0).

### 2.2 Detection model training and testing

#### 2.2.1 High-resolution dataset models

Various pretrained versions of two state-of-the-art one-stage object detection model families [YOLO11 by Jocher et al. (2025) and YOLO-NAS by Aharon et al. (2024)] were retrained (fine-tuning) in Python until model convergence using the internal high-resolution (pixel size: 5 *µm*) training and validation datasets. An overview of the studied model versions and corresponding Python libraries is shown in **Table 1**. To improve the overall generalization of each model, all default data augmentation techniques of both Python libraries were used during training. Considering the proof-of-concept purpose of this study, only the main model hyperparameters (e.g. number of epochs, batch size, initial/warm-up learning rate and epochs, etc.) were adjusted in between (re)training iterations in order to obtain the best configuration per model version (see **Supplementary Table S1** and **Supplementary Table S2** in the **Supplementary Material File**). Subsequently, all best model versions were tested on both the internal and external high-resolution test datasets in order to compare both the performance and generalization, relative to the other model versions.

**Table 1:** Overview of the studied detection model versions (Aharon et al., 2024; Jocher et al., 2025) that were trained on the high-resolution (pixel size: 5 *µm*) internal dataset.

Model type	Version	Size (M parameters)	Developer (year)	Python package (version)
YOLO11	YOLO11n YOLO11s YOLO11mYOLO11lYOLO11x	2.6 9.4 20.125.356.9	Ultralytics (2025) Ultralytics (2025) Ultralytics (2025) Ultralytics (2025) Ultralytics (2025)	ultralytics (v8.3.58) ultralytics (v8.3.58) ultralytics (v8.3.58)ultralytics (v8.3.58)ultralytics (v8.3.58)
YOLO-NAS	YOLO-NAS-S YOLO-NAS-MYOLO-NAS-L	19.0 51.166.9	Deci AI, Inc. (2024) Deci AI, Inc. (2024)Deci AI, Inc. (2024)	super-gradients (v3.6.1) super-gradients (v3.6.1)super-gradients (v3.6.1)

The following test performance metrics (macro-averaged and/or classwise) were extracted for each model version. The corresponding formula to calculate each of these metrics were added to the **Supplementary Material File** (**Equations S1–S6**):

- precision@50: The correctness of the model detections, calculated at an IoU threshold of 50%.
- recall@50: The ability to detect all present objects, calculated at an IoU threshold of 50%.
- F1@50: The harmonic mean of the detection precision and recall, calculated at an IoU threshold of 50%.
- AP@50: The average precision or the area under the precision-recall curve for a given detection class, calculated at an IoU threshold of 50%.
- mAP@50: The mean average precision (mAP) or average AP@50 over all detection classes, calculated at an IoU threshold of 50%.
- mAP@50:95: The average of the mAP scores, calculated at various IoU thresholds ranging from 50% to 95%, with a step size of 5%.

All models were tested using the built-in Python package functions or dedicated code if needed. The optimal overall test confidence threshold was obtained from the (smoothed) macro-averaged F1@50 confidence curve, by taking the (lowest) confidence score resulting in the maximum F1@50 value. An overview of all other hyperparameter values that were used during model testing can be found in **Supplementary Table S3** and **Supplementary Table S4** in the **Supplementary Material File**. Considering the intended application (automated species-level monitoring in greenhouse horticulture), the authors arbitrarily defined a minimum practical feasibility threshold of 70% for the macro-averaged mAP@50, F1@50, precision@50 and recall@50. However, as this threshold will be highly crop and grower-specific, readers/future users are encouraged to adjust it according to their specific requirements.

To support the interpretation of the model performances, the confusion matrices were also generated. Furthermore, the Gradient-weighted Class Activation Maps (Grad-CAMs) of the last C3k2 model block were created for the smallest, yet practically feasible YOLO11 model version. This was done for the complete external test dataset and an additional mosaic patch containing all four studied pest species (originating from the external test dataset) using the same hyperparameters as during model testing. The obtained Grad-CAMs allowed for a superficial comparison between the most decisive pest features/regions used by the model and the key morphological species characteristics that are listed in the literature (European and Mediterranean Plant Protection Organization, 2004; Mound et al., 2025).

#### 2.2.2 Minimum resolution research

To study the influence of the image (pixel) resolution and model complexity on the model’s performance and generalization, both a small (YOLO11n) and big (YOLO11x) detection model were retrained (see **Supplementary Table S9** and **Supplementary Table S10** in the **Supplementary Material File**) and tested (see **Supplementary Table S11** and **Supplementary Table S12** in the **Supplementary Material File**) on each of the reduced-resolution datasets. The YOLO11 model type (Jocher et al., 2025) was used for this research due to the faster training/testing process and user-friendly Python library.

Based on the earlier defined practical feasibility threshold of 70% (or other value chosen by the reader/future user) and the obtained test metrics of each reduced-resolution model, the corresponding minimum required image resolution/maximum pixel size for species-level detection could subsequently be determined. Finally, to study the practical feasibility of stand-alone automated species-level detection traps, this value was translated into various potential minimum required photography setups. This was done using the technical specifications of the selected cameras/lenses (see **Supplementary Table S17**) and **Equations S7–S9** in the **Supplementary Material File**. As the horizontal and vertical angles of view were not listed in the official data sheets of the iPhone 16 Pro and the Sony *α*7R III, these values were manually calculated using the diagonal angles of view, aspect ratios (width:height) of respectively 4:3 and 3:2, and **Equations S10, S11** in the **Supplementary Material File**.

#### 2.2.3 Technical specifications

Model training, validation and testing were locally executed on a workstation in Python (v3.8.19) using the following main libraries: ultralytics (v8.3.58), super-gradients (v3.6.1) and tensorboard (v2.18.0). The workstation consisted of one NVIDIA RTX A5000 GPU (NVIDIA Corporation, Santa Clara, CA, USA | CUDA version: v12.2.140) and two Intel Xeon Gold T CPUs (Intel Corporation, Santa Clara, CA, USA).

## 3 Results

### 3.1 Dataset acquisition

#### 3.1.1 High-resolution datasets

Over the course of several months, dozens of YSTs were collected, photographed, processed and labelled for each pest type, resulting in a heterogeneous internal dataset of 5105 image patches (see **Table 2**) and an external test dataset of 246 image patches (see **Table 3**). Due to a limited contamination of the *T. vaporariorum* cultivation with *F. occidentalis* individuals, some of the prior image patches contained both insect species. Because of this, the sum of the individual image patches per patch type does not equal the listed total (sub)dataset sizes of the training set, test set and total dataset in **Table 2**. This also counts for the external dataset (**Table 3**) as often multiple pest species occurred on the same image patch. However, for the latter dataset this was intended (mixed test dataset). Lastly, as all background images originated from the same sticky traps as the four studied pest species and ‘other insects’ datasets, also the listed total amount of unique sticky trap sides per (sub)dataset does not equal the sum of the individual unique trap sides per image patch type, in both tables.

**Table 2:** Detailed composition of the high-resolution (pixel size: 5 *µm*) internal dataset, consisting of yellow sticky trap image patches.

Image/label type	Training set (60%)		Validation set (20%)		Test set (20%)		Total dataset (100%)		
	Labels	Image patches	Labels	Image patches	Labels	Image patches	Labels	Image patches	Unique sticky trap sides (A+B)
*F. occidentalis* (class 1)	548	450	215	155	237	182	1,000	787	23 (11 + 12)
*E. americanus* (class 2)	600	567	192	188	208	202	1,000	957	15 (8 + 7)
*B. tabaci* (class 3)	626	529	189	168	185	153	1,000	850	10 (5 + 5)
*T. vaporariorum* (class 4)	587	297	247	113	166	85	1,000	495	16 (7 + 9)
other insects (no class)	–	621	–	194	–	185	–	1,000	34 (29 + 5)
yellow background (BG - no class)	–	146	–	50	–	59	–	255	64 (38 + 26)
printed grid (BG - no class)	–	149	–	50	–	53	–	252	41 (41 + 0)
reflections (BG - no class)	–	158	–	45	–	53	–	256	63 (36 + 27)
identification sticker (BG - no class)	–	62	–	22	–	21	–	105	35 (18 + 17)
sticky trap mount (BG - no class)	–	90	–	35	–	33	–	158	58 (31 + 27)
TOTAL	2,361	3,062^a^	843	1,020	796	1,023^a^	4,000	5,105^a^	88 (56 + 32)^b^

The following abbreviations are used: side A = printed grid front side; side B = non-printed back side; BG = background image.

a Due to a limited contamination of the *T. vaporariorum* cultivation with *F. occidentalis* individuals, some of the prior image patches contained both insect species. Because of this, the sum of the individual image patches per patch/label type does not equal the listed total (sub)dataset sizes of the training set, test set and total dataset.

b As all background images originated from the same sticky traps as the four studied pest species and ‘other insects’ datasets, the listed total amount of unique sticky trap sides does not equal the sum of the individual unique sticky trap sides per image/label type.

**Table 3:** Detailed composition of the high-resolution (pixel size: 5 *µm*) external dataset, consisting of yellow sticky trap image patches.

Image/label type	Test set (100%)		Total dataset (100%)		
	Labels	Image patches	Labels	Image patches	Unique sticky trap sides (A+B)
*F. occidentalis* (class 1)	54	47	54	47	8 (4 + 4)
*E. americanus* (class 2)	54	54	54	54	7 (4 + 3)
*B. tabaci* (class 3)	54	51	54	51	4 (2 + 2)
*T. vaporariorum* (class 4)	54	36	54	36	4 (2 + 2)
other insects (no class)	–	54	–	54	8 (4 + 4)
yellow background (BG - no class)	–	12	–	12	8 (4 + 4)
printed grid (BG - no class)	–	12	–	12	4 (4 + 0)
reflections (BG - no class)	–	12	–	12	8 (4 + 4)
identification sticker (BG - no class)	–	5	–	5	5 (3 + 2)
sticky trap mount (BG - no class)	–	8	–	8	7 (3 + 4)
TOTAL	216	246^a^	216	246^a^	8 (4 + 4)^b^

The following abbreviations are used: side A = printed grid front side; side B = non-printed back side; BG = background image.

a As some image patches contained multiple thrips/whitefly individuals, the sum of the individual patches per image/label type does not equal the listed total (sub)dataset size.

b As all background images originated from the same sticky traps as the four studied pest species and ‘other insects’ datasets, the listed total amount of unique sticky trap sides does not equal the sum of the individual unique sticky trap sides per image/label type.

#### 3.1.2 Reduced-resolution datasets

The resolution downscaling process successfully resulted in eight different reduced-resolution versions (theoretical pixel size - TPS: 5 *µm* up to 640 *µm*) of both the internal and external datasets (see **Supplementary Figure S2** in the **Supplementary Material File**).

### 3.2 Detection model training and testing

#### 3.2.1 High-resolution dataset models

**Figure 3:**
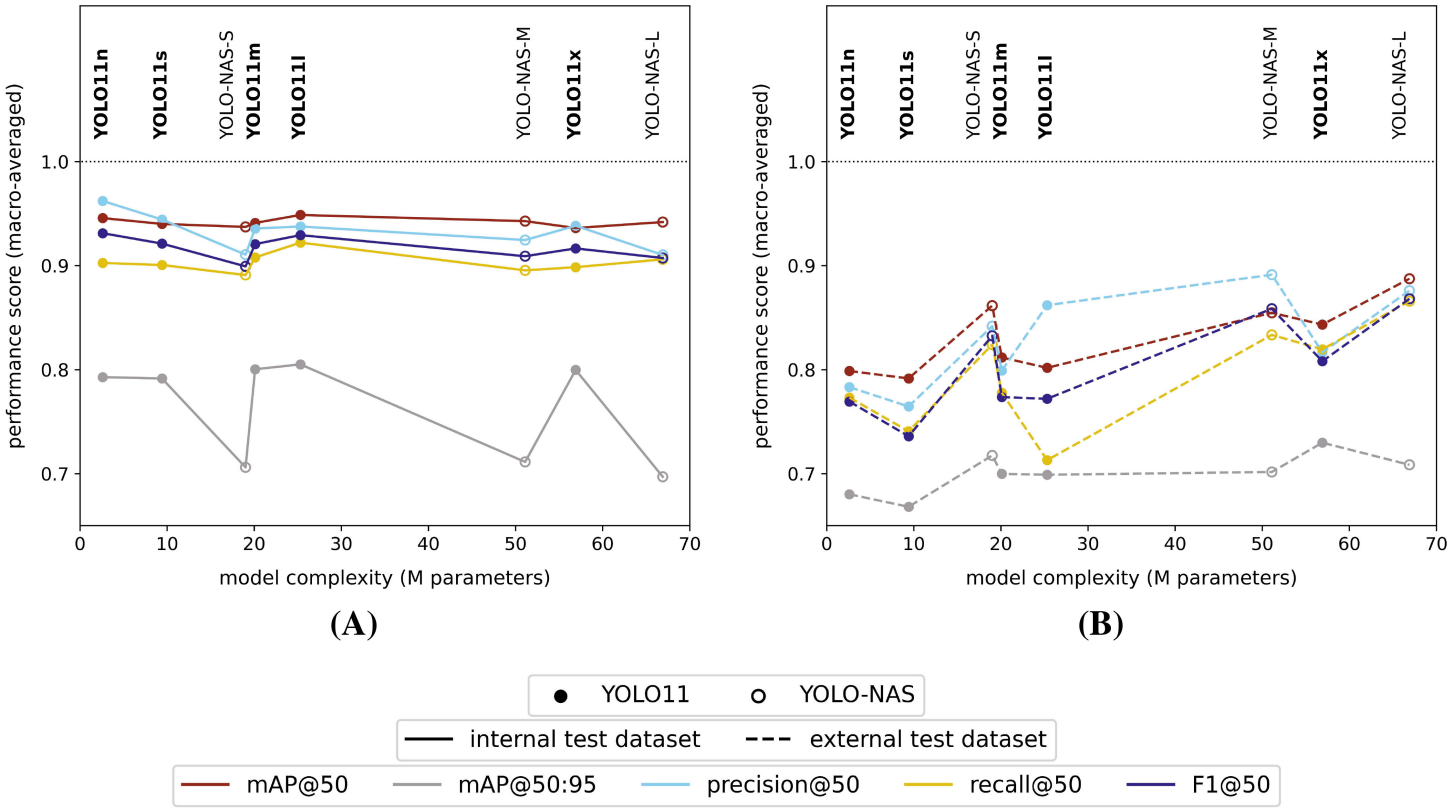
Macro-averaged high-resolution (pixel size: 5 *µm*) internal **(A)** and external **(B)** test dataset performance scores for each of the studied model versions, trained on the internal high-resolution image dataset.

All studied model versions provided comparable general performance scores (mAP@50 and F1@50) for the high-resolution (pixel size: 5 *µm*) internal test dataset of ≥ 90%, no matter the used model type, version or general complexity (**Figure 3A**). However, the mAP@50:95 performance score for the internal test dataset was consistently lower for the YOLO-NAS models compared to the YOLO11 models (ΔmAP@50:95 = 8% - 11%).

All models clearly performed worse on the external test dataset (**Figure 3B**), but did show an increasing performance towards higher model complexities. Furthermore, the YOLO-NAS models tended to better generalize to the unseen external test dataset (mAP@50: 85% - 89% | F1@50: 83% - 87%) compared to the YOLO11 models (mAP@50: 79% - 84% | F1@50: 74% - 81%). In general, a performance drop between both test datasets of approximately 10% - 20% and < 10% was observed, respectively, for the YOLO11 and YOLO-NAS model architectures for all performance metrics with an IoU ≥ 50% (mAP@50, precision@50, recall@50 and F1@50). However, the drop in mAP@50:95 scores between both test datasets was less pronounced for the YOLO11 models (approx. 10%), while almost non-existing for the YOLO-NAS models.

**Figure 4:**
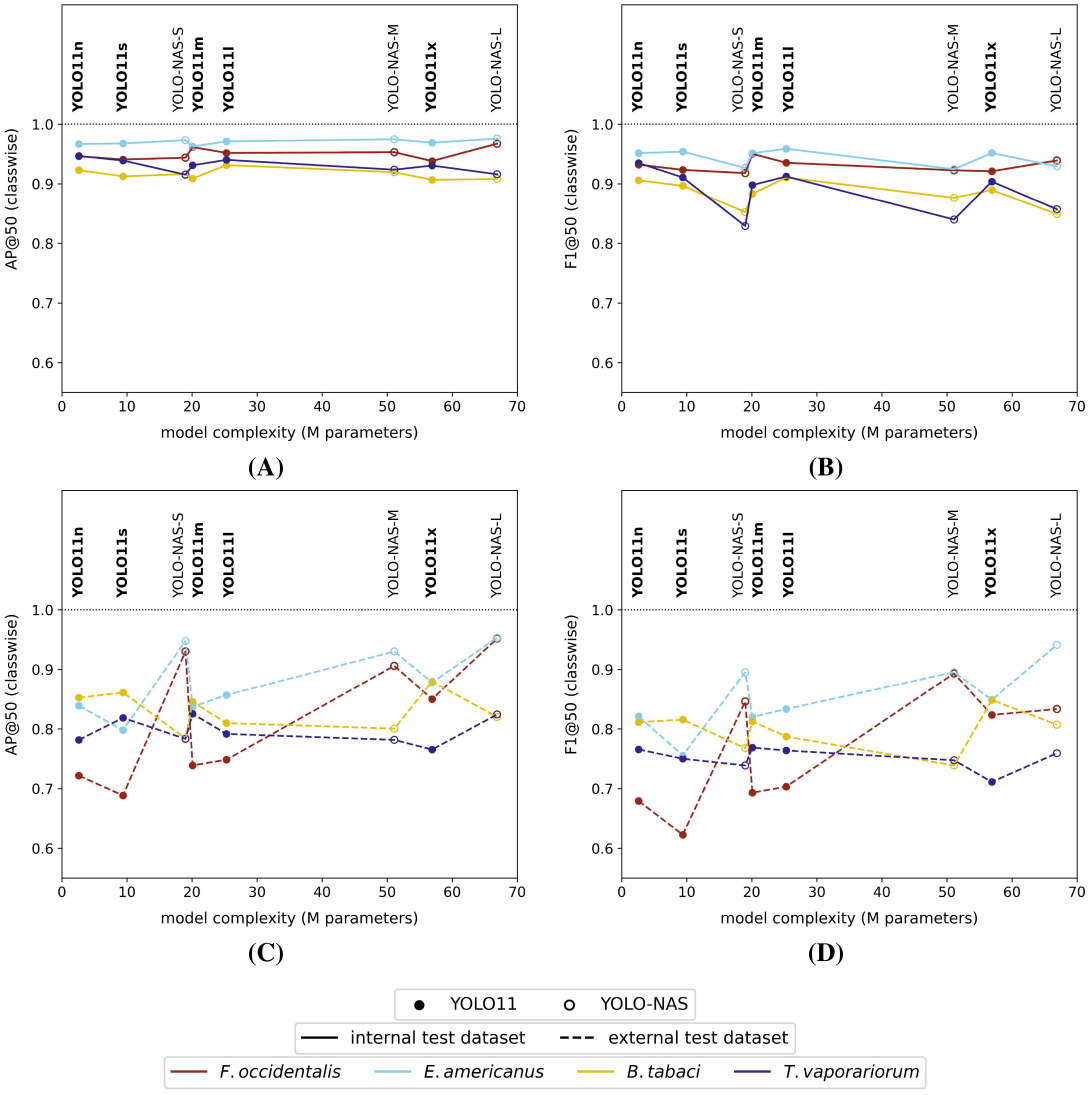
Classwise **(A)** AP@50 and **(B)** F1@50 performance scores on the high-resolution (pixel size: 5 *µm*) internal test dataset and classwise **(C)** AP@50 and **(D)** F1@50 performance scores on the high-resolution external test dataset for each of the studied model versions. All models were trained on the internal high-resolution image dataset.

When studying the classwise model performances on the high-resolution internal test dataset, all model versions generally performed better (highest mAP@50 and F1@50) for the detection of thrips (*E. americanus* and *F. occidentalis*) compared to the detection of both whitefly species (*T. vaporariorum* and *B. tabaci*) (**Figures 4A, B**). However, it should be noted that the absolute differences between the best and worst performing classes were rather limited for all tested model versions (ΔAP@50: 4% - 7% | ΔF1@50: 4% - 10%). The corresponding precision@50 and recall@50 plots for each tested model were visualized, respectively, in **Supplementary Figure S4C** and **Supplementary Figure S4D** in the **Supplementary Material File**.

However, considering the external test dataset (**Figures 4C** and **4D**), the overall order of the best performing classes was different for the YOLO11 models, compared to the equivalent model versions, tested on the internal dataset. The general performance drop was largest for the smallest YOLO11 models (YOLO11n and YOLO11s), now performing best on *B. tabaci* (ΔAP@50: -7% and -5% | ΔF1@50: -10% and -8% | relative to the internal test dataset performance) and worst on *F. occidentalis* (ΔAP@50: -23% and -25% | ΔF1@50: -25% and -30% | relative to the internal test dataset performance). However, this effect was reduced when using more complex model versions. Apart from a performance drop compared to the internal test dataset, the order of best performing pest classes did not really change for the YOLO-NAS models. The corresponding general performance drop was clearly most significant for both whitefly species (ΔAP@50: -9% to -14% | ΔF1@50: -4% to -14% | relative to the internal test dataset performance). Once again, the precision@50 and recall@50 plots were added to the **Supplementary Material File** (**Supplementary Figure S5C**, **Supplementary Figure S5D**).

Within the YOLO-NAS model series, the YOLO-NAS-L model performed best on the external test dataset, resulting in macro-averaged and classwise performance scores (IoU ≥ 50%) of respectively > 85% and ≥ 75%. For the YOLO11 model series, the largest YOLO11x version performed best on the external test dataset, resulting in 5% - 6% lower macro-averaged performance scores (IoU ≥ 50%) compared to the best YOLO-NAS model (YOLO-NAS-L).

**Figure 5:**
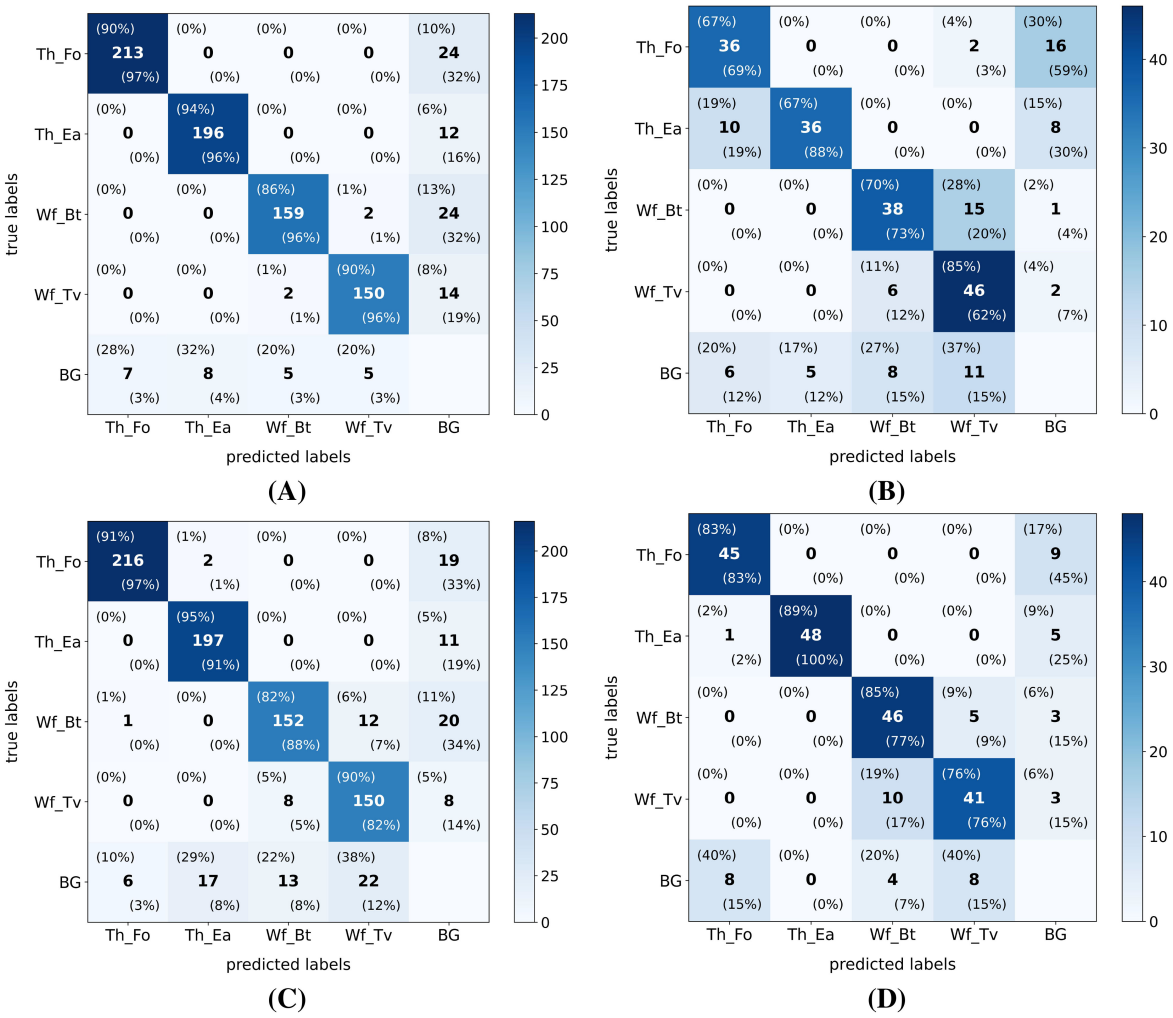
Exemplary confusion matrices (IoU ≥ 50%) after testing the smallest (YOLO11n) and biggest (YOLO-NAS-L) model versions on both the internal and external high-resolution (pixel size: 5 *µm*) datasets. Both model versions were trained on the high-resolution internal dataset. Both the absolute (center value) and normalized (top left: ground-truth normalized | bottom right: prediction normalized) values are added to each cell. The following model version-test dataset combinations are plotted: **(A)** YOLO11n - internal test dataset; **(B)** YOLO11n - external test dataset; **(C)** YOLO-NAS-L - internal test dataset and **(D)** YOLO-NAS-L - external test dataset. Hereby the following label abbreviations were used: Th_Fo: *F*. *occidentalis* (thrips); Th_Ea: *E*. *americanus* (thrips); Wf_Bt: *B*. *tabaci* (whitefly); Wf_Tv: *T. vaporariorum* (whitefly) and BG: background/other insects.

The previously described similar performance of all model versions on the internal test dataset and better generalization to the external test dataset by the YOLO-NAS models are also clearly visible in the confusion matrices (IoU ≥ 50%) of the smallest (YOLO11n) and largest (YOLO-NAS-L) tested model versions (see **Figure 5**). Although most distinct for the smallest YOLO11n model, the proportion of complete misses (pest insect predicted as background) was in both models higher for the thrips classes in the external test dataset, compared to the internal test dataset. However, regarding the whitefly detections, the percentage of complete misses generally dropped while species-level misclassifications (*B. tabaci* <=> *T. vaporariorum*) significantly increased in the external dataset. Lastly, the proportion of false detections (background detected as pest insect) was also higher in the external test dataset for both model versions, while being most pronounced for the YOLO11n model.

The Grad-CAMs of the smallest, yet practically feasible, high-resolution model version (YOLO11n), indicated that the model mainly focused on the (upper part) of the abdomen of both thrips species, when visible. When occluded, the model also focused on the head and antennae. Regarding the studied whitefly species, the model generally focused on the yellow body and darker white/transparent zones of the wings. An exemplary overview of the classwise YOLO11n Grad-CAMs was added in **Supplementary Figure S3** of the **Supplementary Material File**.

#### 3.2.2 Minimum resolution research

Due to the rather time-consuming neural architectural search (NAS) process during the YOLO-NAS model training and the user-friendly ultralytics Python library, in the end the YOLO11 model architecture was used for the minimum resolution research.

**Figure 6:**
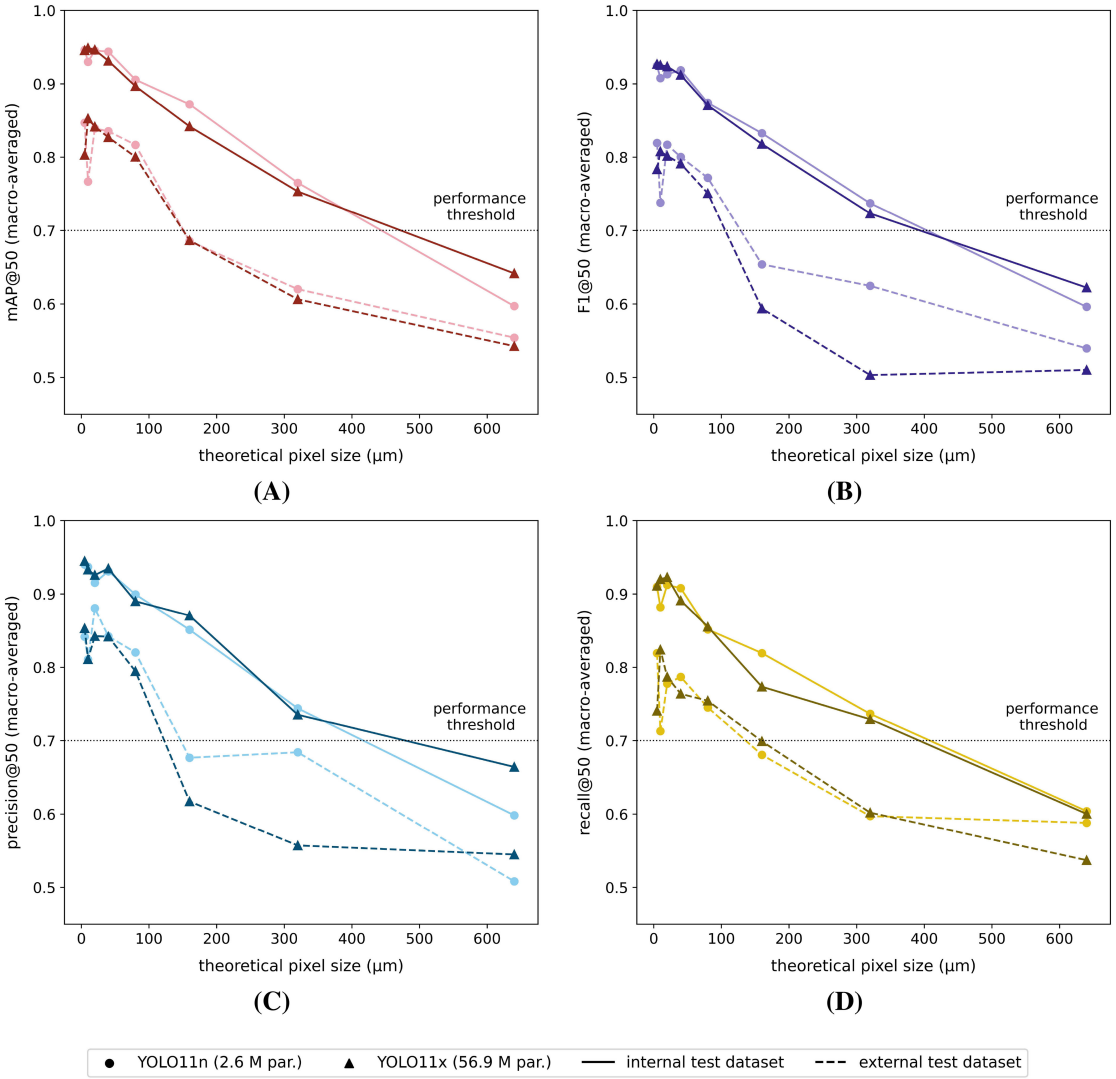
Macro-averaged internal/external test dataset **(A)** mAP@50, **(B)** F1@50, **(C)** precision@50 and **(D)** recall@50 performance scores for each of the YOLO11n and YOLO11x model versions, trained on the corresponding reduced-resolution internal datasets (theoretical pixel size: 5 *µm* - 640 *µm*). The black dotted line represents the arbitrarily defined minimum required practical feasibility threshold of 70% for greenhouse horticultural applications.

Once again, all tested models performed worse on the external test dataset, compared to the internal test dataset (**Figure 6**). Furthermore, the differences were generally larger for lower image resolutions (= larger pixel sizes). Both model types showed similar macro-averaged performance (mAP@50, F1@50, precision@50 and recall@50) on the internal test dataset. The model performance dropped almost linearly with increasing pixel sizes. When tested on the external dataset, both model types also performed quite similarly for the smaller (theoretical) pixel size datasets, while for larger pixel size datasets, the YOLO11n models performed generally better. Both the mAP@50, F1@50 and precision@50 scores showed a noticeable, non-linear drop at pixel sizes > 80 *µm*. In general, none of the reported macro-averaged performance measures (IoU ≥ 50%) dropped below 50% for the full range of studied (theoretical) pixel sizes and model architectures.

**Figure 7:**
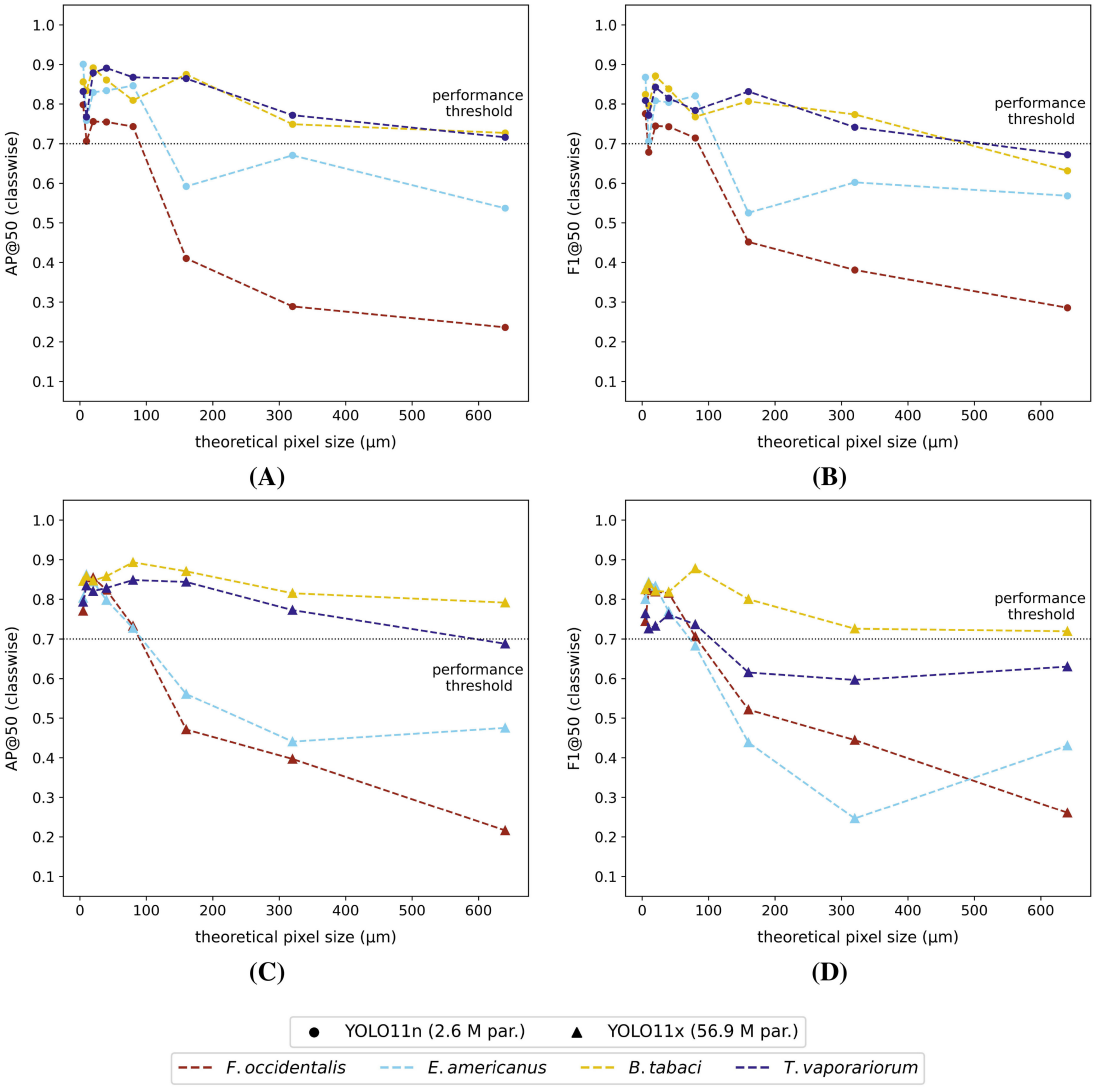
Classwise external dataset **(A)** AP@50 and **(B)** F1@50 performance scores for each of the YOLO11n models and classwise external dataset **(C)** AP@50 and **(D)** F1@50 performance scores for the YOLO11x models. All models were trained on the corresponding internal reduced-resolution image datasets (theoretical pixel size: 5 *µm* - 640 *µm*). The black dotted line represents the arbitrarily defined minimum required practical feasibility threshold of 70% for greenhouse horticultural applications.

Although the macro-averaged performances of both model types were quite comparable for the external test dataset (**Figure 6**), the classwise external dataset performances of the YOLO11x models were generally inferior across all dataset resolutions (**Figure 7**, **Supplementary Figures S8, S9**). In general, the classwise detection performances (AP@50 and F1@50) on the external dataset were for thrips, and in particular *F. occidentalis*, strongly affected by the image resolution, showing a steep performance drop for pixel sizes > 80 *µm* for both model types. The performance for both whitefly species (*B. tabaci* and *T. vaporariorum*) on the other hand remained much more stable with increasing pixel sizes. The classwise external test dataset precision@50 and recall@50 were plotted in the **Supplementary Material File** for each of the reduced-resolution YOLO11n (**Supplementary Figure S8C**, **Supplementary Figure S8D**) and the YOLO11x models (**Supplementary Figure S9C**, **Supplementary Figure S9D**). For completeness, also the classwise performance (IoU ≥ 50%) on the internal test dataset of all tested model versions/image resolutions were visualized in the **Supplementary Material File** (**Supplementary Figure S6**, **Supplementary Figure S7**).

The internal test dataset performances of both the 5 *µm* reduced-resolution YOLO11n (**Supplementary Table S13**) and YOLO11x models (**Supplementary Table S14**) were almost identical to those of the corresponding high-resolution dataset models (ΔmAP@50: 0% - 1% | ΔF1@50: 1% | Δ*max_classwise_*@50: 3% | **Supplementary Table S5**). However, when tested on the external test dataset, the reduced-resolution 5 *µm* YOLO11n model (**Supplementary Table S15**) performed better than the high-resolution dataset YOLO11n model (ΔmAP@50: 5% | ΔF1@50: 5% | Δ*max_classwise_*@50: 17% | **Supplementary Table S7**). Meanwhile, the opposite behaviour was observed for the YOLO11x model version (**Supplementary Table S7**, **Supplementary Table S16** | ΔmAP@50: 4% and ΔF1@50: 3% | Δ*max_classwise_*@50: 13%).

Considering the (arbitrarily defined) minimum macro-averaged practical feasibility threshold of 70% (IoU ≥ 50%), the minimum required (theoretical) pixel resolution for species-level detection was identified as 80 *µm* (theoretical pixel size - TPS: ≤ 80 *µm*) for both the YOLO11n and YOLO11x models (**Figure 6**). Furthermore, almost all classwise AP@50 and F1@50 scores also exceeded this threshold at TPS ≤ 80 *µm* (**Figure 7**). By contrast, classwise precision@50 and recall@50 scores did not always comply with the 70% threshold (see **Supplementary Figures S8, S9**) for TPS ≤ 80 *µm*.

In **Table 4**, various alternative sticky trap photography setups, corresponding to the minimum (theoretical) required pixel resolution for the YOLO11n and YOLO11x model versions (TPS: 80 *µm*), were compared with the camera system used in this research.

**Table 4:** Theoretical minimum required photography setups suited for species-level detection of the studied thrips and whitefly species (theoretical pixel size - TPS: 80 *µm*) for the used high-resolution DSLR camera (bold font), recent smartphone cameras, recent action camera, and some widely used low-budget camera modules.

Sensor type + lens	Sensor quality	Minimum focus distance	Angle of view (hor. and ver.)	Max. effective^a^ working distance (TPS: 80 *µm*)	Images per sticky trap	Approximate unit cost^b^
Raspberry Pi camera module 2	8 MP	10 cm	H: 62.2° V: 48.8°	21 cm	1	< € 50
Raspberry Pi camera module 3	11.9 MP	10 cm	H: 66° V: 41°	28 cm	1	< € 50
Arducam Pi Hawk-eye	64 MP	8 cm	H: 72° V: 54.6°	52 cm	1	< € 100
Samsung Galaxy A16 LTE (main camera)	50 MP	10 cm	H: 67.6° V: 53.8°	48 cm	1	< € 150
GoPro HERO13 Black + macro lens module (16:9 - linear mode)	27.13 MP	11 cm	H: 87° V: 56°	29 cm	1	< € 500
iPhone 16 Pro (ultra wide camera)	48 MP	2 cm	H: 108° V: 92°	23 cm	1	< € 1,250
**Sony *α*7R III + FE 50mm F2.8 macro lens**	**42.4 MP**	**16 cm**	**H: 40°** **V: 27°**	**87 cm**	**1**	**< € 2,250**

All listed cameras are equipped with auto-focus.

a Rounded down to the nearest cm.

b Approximate unit cost (rounded up to the closest multiple of €50) at the time of submission of the manuscript.

The used equations and references to the technical specifications/prices can be found in **Supplementary Equations S7**-**S11** and **Supplementary Table S17**, respectively, of the **Supplementary Material File**.The bold values correspond to the imaging setup that was used in this research.

## 4 Discussion

### 4.1 Dataset acquisition

#### 4.1.1 High-resolution datasets

The image acquisition and processing methods resulted in a standardized and balanced high-resolution (pixel size: 5 *µm*) internal dataset, consisting of 1,000 labels per studied pest species on wet glue YSTs and relevant background images. Although the standardized light conditions and high-quality DSLR camera setup are not practically/economically feasible for standalone smart traps in greenhouse horticulture, our standardized dataset did allow an objective evaluation of the species-level monitoring concept. Furthermore, our dataset can be easily converted to various other, more realistic scenarios such as fluctuating light conditions/spectra and camera quality/type using various image processing techniques.

As the morphology (shape, size and colour) of body structures can vary within pest species depending on local environmental conditions, population densities and species genetics (Higgins and Myers, 1992; Mound, 2005; Riley et al., 2011), future datasets should incorporate as much of these variables in order to increase the model generalization. Furthermore, the dataset in this study was limited to one brand of wet glue YSTs, while in practice various manufacturers, trap colours and glue types (wet and dry) are used, depending on the targeted pest insect and farming conditions/location. As suggested by Ong and Høye (2025), to further enhance model generalization across different trap colours without significant performance loss, the training dataset should ideally comprise insect images captured on transparent sticky traps. To overcome the limited chromotropic attraction and lack of background contrast of these traps, the addition of a coloured background behind the transparent trap is suggested to collect the training dataset.

Although theoretically possible by altering the standardized, high-resolution dataset, extending the training dataset with real images taken by various sensor types (e.g. DSLR camera, smartphone camera, action camera, stand-alone camera module, etc.) and lighting conditions could also help the model to better generalize to real-world conditions and mixed imaging setups (Ong and Høye, 2025). Finally, the practical implementation and image acquisition protocol using smaller, standalone camera sensors will most likely also be more feasible compared to the standardized DSLR camera setup that was used in this research.

#### 4.1.2 Reduced-resolution datasets

The high-resolution dataset (pixel size: 5 *µm*) and the proposed digital resolution downscaling process allowed for a fast and easy creation of various reduced-resolution datasets (TPS: 5 *µm* - 640 *µm*). However, the main limitation of this method was the fact that this only resulted in digitally reduced resolution datasets which will to some extend differ (e.g. presence/absence of specific artifacts, distortions, noise, etc.) from images originally taken at lower resolutions. The latter effect is expected to be more significant for lower resolutions (see **Supplementary Figure S2** - TPS: 320 *µm* and 640 *µm*) as the difference in pixel resolutions is larger compared to the original high-resolution dataset. Furthermore, DSLR camera images provide the best image quality, where other camera systems with reduced sensor sizes (e.g. smartphones and low-cost camera modules) suffer from more noise in the images taken. The latter images are also often digitally optimized to provide better results, increasing the number of artifacts that become visible when zoomed in. Therefore, in future research, lower-resolution datasets should ideally be obtained using lower-resolution camera systems and compared to the reduced-resolution datasets of this research.

### 4.2 Detection model training and testing

#### 4.2.1 High-resolution dataset models

##### 4.2.1.1 Model performance and generalization

Considering the (arbitrarily defined) minimum macro-averaged practical feasibility threshold of 70% at IoU ≥ 50%, species-level thrips and whitefly detection could be considered as feasible for each of the tested high-resolution dataset (pixel size: 5 *µm*) models and both test datasets. The reduced, yet still feasible generalization to the external test dataset of all tested models was most likely caused by the rather limited size (1,000 labels/pest species) and heterogeneity (unique sticky trap sides) of the obtained internal high-resolution dataset. Extending the training dataset with more unique image patches could potentially further improve the models’ generalizations. Furthermore, small differences in dataset quality/composition and the limited external test dataset size (246 image patches <=> 1023 image patches) could also have played a role.

The observed better generalization to the external test dataset of more complex model versions supports the findings of Hu et al. (2021), Neyshabur et al. (2014) and Novak et al. (2018). The reason why this relative advantage of more complex models could not be observed for the internal test dataset performances, was most likely due to the more similar image patches during training and testing (same original sticky traps | same acquisition dates), compared to the external test dataset (independent dataset | different acquisition date). As the models do not need to handle (subtle) differences in acquisition settings and dataset quality, the benefit of using more complex models will therefore be limited compared to the increased risk of overfitting.

The lower observed mAP@50:95 scores of the YOLO-NAS models on the internal test dataset were most likely caused by the best model selection method of the super-gradients Python library. For the YOLO-NAS models, this selection was based on the mAP@50:75 validation metric, while for the YOLO11 models (ultralytics Python package) the model fitness (weighted average: mAP@50 = 10% and mAP@50:95 = 90%) parameter was used. Furthermore, Ali and Zhang (2024) reported general YOLO-NAS detection struggles for occluded objects which could also have played a role. The better generalization of the YOLO-NAS models to the external test dataset could be linked to the intrinsic training process. During YOLO-NAS training, both the model architecture and weights are optimized in terms of computational resources and inference time, while limiting the impact on model accuracy (Ali and Zhang, 2024; Terven et al., 2023). As this optimization process generally results in less complex models, the finally obtained YOLO-NAS models might be less prone to overfitting to the original dataset, therefore boosting their generalization.

Regarding the classwise general performance on the internal high-resolution dataset, all models scored best for the detection of thrips (in particular *E. americanus*) and worst for the detection of whiteflies (mostly *B. tabaci*), although the absolute differences between the best and worst performing classes were limited. However, when testing on the external dataset, the smallest YOLO11 models (YOLO11n and YOLO11s) performed best for the detection of *B. tabaci* and worst for the detection of *F. occidentalis*. Based on the reported confusion matrices, this drop in thrips performance was caused by an increase in complete misses (thrips detected as background). As this effect was reduced when using more complex YOLO11 model versions, we suggest that (subtle) differences in the image acquisition method and lower image quality (less focussed) of the external dataset might have caused this. All YOLO-NAS models performed best for the detection of thrips on the external dataset and resulted in lower general classwise performance drops between the internal and external datasets compared to the YOLO11 models. The increased ratio of YOLO-NAS-L whitefly misclassifications in the external test dataset, as seen in the reported confusion matrices, can most likely also be attributed to differences in dataset composition, especially insect residence times (IRTs), and lower image quality (less focused).

As almost all tested high-resolution models output both macro-averaged and classwise performance metrics (IoU ≥ 50%) of respectively > 70% and > 60% on the external dataset, species-level detection using the proposed high-resolution (pixel size: 5 *µm*) imaging setup seemed to be possible for the studied pest species. Furthermore, although model performance increased with model complexity, species-level detection also seemed to be possible using the smallest tested model version (YOLO11n), resulting in overall/classwise performance metrics of > 65% (IoU ≥ 50%) for the external test dataset. Furthermore, the Grad-CAMs of this model showed overlap between the class-specific focus regions of the model and the key morphological characteristics of each species, described in the literature (European and Mediterranean Plant Protection Organization, 2004; Mound et al., 2025). Thrips focus areas were mainly concentrated on the abdomen, containing the differently coloured striping pattern and abdominal colour. Key focus areas of the studied whitefly species were the body and dark white/transparent zones of the wings. Differences in species body colour were also described in the literature (European and Mediterranean Plant Protection Organization, 2004). The fact that the model seemed to focus on the transparency of the wings, could be explained by the fast (IRTs: less than seven days) decaying wings of *B. tabaci*, as described by Böckmann et al. (2021). This observation was also shared by the authors while handling the datasets (data not shown). As the whitefly pests in our datasets had IRTs of several days up to several months, the trained models might possibly fail when applied to YSTs containing fresh whiteflies. However, this issue can most likely be tackled by extending the training datasets with image patches of fresh individuals.

Furthermore, since none of the high-resolution models performed consistently worse for the detection of *E. americanus* (IRTs: approx. one year) in the external dataset compared to the other detection classes (IRTs: several days to several months), this showcases the potential of species-level detection, even when using older YSTs. However, it should be noted that the decay of *E. americanus* over time was rather limited in our dataset (data not shown), which could explain this observation. Differences in IRTs between the training and testing datasets are namely known to negatively impact the detection performance, as well as insect decay in general (Böckmann et al., 2021). Enriching the dataset with images featuring a wide range of IRTs and varying stages of decay would likely enhance the models’ generalization/robustness to these factors.

##### 4.2.1.2 Comparison with existing research

The best overall detection results (in this study) for the high-resolution (pixel size: 5 *µm*) external dataset were obtained using the YOLO-NAS-L model version (mAP@50: 89% | F1@50: 87%) and YOLO11x model version (mAP@50: 84% | F1@50: 81%), clearly complying with the earlier defined macro-averaged practical feasibility threshold of 70% (IoU ≥ 50%). Compared to the only reported species-level detection models on sticky trap images in the literature - developed by Niyigena et al. (2023) using 17 *µm* pixel resolution and achieving macro-averaged mAP@50 scores of 91% (reflectance dataset) up to 97% (transmittance dataset) for detecting *S. dorsalis* and other thrips species (included as one class) - all high-resolution models (pixel resolution: 5 *µm*) that were tested in this study performed similarly on the internal test dataset, with mAP@50 scores ranging from 94% to 95%. However, as Niyigena et al. (2023) focused on different pest species, detected only one species at the species level, and lacked an external test dataset, a more profound comparison could not be made. In addition, as the models of Niyigena et al. (2023) were trained and tested using images, pre-annotated with differently coloured markers per class prior to image acquisition, one could question the generalization of the reported models. Insect detection could, to some extent, rely on the presence or absence of these coloured circles rather than on the insect features themselves. Compared to the best overall performing *T. vaporariorum* and *B. tabaci* adult detection model of Gutierrez et al. (2019) on internal test dataset plant images (precision: *B. tabaci* = 34% and *T. vaporariorum* = 72%), all high-resolution models in this research performed better on the internal test dataset (precision: *B. tabaci* = 88% - 96% and *T. vaporariorum* = 80% - 96%). However, considering the different type/resolution of input images and dataset size, no further comparison could be made.

##### 4.2.1.3 Future directions

To our knowledge, this is the first reported thrips/whitefly species-level detection system using standard RGB (yellow) sticky trap images and YOLO-based detection models. Furthermore, the results indicated that it was very successful to discriminate at the species level (mAP@50: 79% - 89% | F1@50: 74% - 87%). Future research should therefore extend this technology to additional types of pest species, sticky traps, model architectures and ambient light conditions (e.g. light intensity and spectral composition). To accelerate progress and support the development of a universal species-level pest monitoring system based on RGB sticky trap images, the creation and use of an open-access database is recommended by the authors.

#### 4.2.2 Minimum resolution research

##### 4.2.2.1 Model performance and generalization

The macro-averaged and classwise performance metrics of all YOLO11n (smallest) and YOLO11x (largest) models were comparable for the internal test dataset and decreased linearly with increasing pixel size. However, when tested on the external datasets, a clear general (mAP@50 and F1@50) performance drop at pixel sizes > 80 *µm* was observed for both model versions. This performance drop was mainly caused by a reduction in thrips species detection performance (particularly for *F. occidentalis*), relative to a slower decaying whitefly species detection performance at lower image resolutions. Despite the larger morphological differences (e.g. colour, body shape, etc.) between the studied thrips species compared to the studied whitefly species, the general lower performance for thrips at lower image resolutions was most likely caused by their relatively smaller body size compared to the studied whitefly species. As a consequence, the number of pixels per individual was lower for these insects, resulting in less cues for the model to base its decision on. Extending the training dataset with more unique image patches and insect morphologies could potentially increase both the classwise thrips and macro-averaged model performances.

In general, the YOLO11n models performed better on the external test dataset at lower pixel resolutions (= larger TPS), compared to the heavily complex YOLO11x models. This showcases that increasing the model’s complexity will not always result in better performance and may even lead to poorer generalization due to overfitting, especially when the size of the training dataset is limited (Jegham et al., 2024). More striking was the fact that none of the macro-averaged performance metrics (IoU ≥ 50%) dropped below 50% for both test datasets, even for the very blurry and pixelated 320 *µm* and 640 *µm* theoretical pixel size datasets. However, this was not the case for the classwise performances on the external test dataset (IoU ≥ 50%), which quickly dropped below 50% for *F. occidentalis* at pixel sizes > 80 *µm*.

Considering the resolution downscaling process, the 5 *µm* reduced-resolution dataset images were not altered compared to the original high-resolution images (see source code of the PIL.Image.resize Python package). Consequently, the observed, though very small, differences in internal test dataset performance scores of the 5 *µm* reduced-resolution and high-resolution dataset YOLO11n and YOLO11x models were probably caused by the different hyperparameters used during training. This could also explain the better generalization to the external test dataset by the 5 *µm* reduced-resolution YOLO11n and original high-resolution dataset YOLO11x models.

##### 4.2.2.2 Minimum required pixel resolution

Based on the (arbitrarily defined) minimum macro-averaged practical feasibility threshold of 70% (IoU ≥ 50%), a minimum required theoretical pixel resolution of 80 *µm* (TPS: ≤ 80 *µm*) could be defined for species-level detection using both the YOLO11n and YOLO11x models. Although the corresponding classwise precision@50 and recall@50 scores did not always exceed this threshold, however, the classwise AP@50 and F1@50 scores generally did. Furthermore, the classwise precision and recall scores can be tweaked by altering the (overall or classwise) detection confidence threshold(s). Consequently, the authors do believe that by further model tweaking, both models’ precision@50 and recall@50 could potentially also exceed/closely approach the defined 70% threshold.

Taking into account this minimum required resolution (TPS: ≤ 80 *µm*), sticky trap species-level detection was subsequently found to be feasible using various alternative imaging setups, such as recent smartphones, action cameras, or even low-cost (< €50 - €100), standalone camera modules such as the Raspberry Pi camera module 2/3 or Arducam Pi Hawk-eye. Moreover, all listed photography setups (see **Table 4**) were able to capture a complete sticky trap in a single image with sufficient resolution and feasible working distances for practical implementation. Furthermore, Ong and Høye (2025) found no significant differences in insect detection model performances when their models were trained on high-quality DSLR, webcam or smartphone camera images of similar resolution, showcasing the potential of low-budget, standalone camera modules for species-level detection. Combined with the limited size and good performance of the YOLO11n model, this really paves the way towards feasible, real-time, automated species-level monitoring of sticky traps for use in greenhouse horticulture.

##### 4.2.2.3 Future directions

Lastly, as the reported reduced-resolution model performances were only theoretically defined, future research should empirically validate these results. Furthermore, also the reported minimum required pixel resolution (80 *µm*) for the YOLO11n and YOLO11x models, should be validated using the proposed (low-cost) camera setups.

## 5 Conclusions

This study demonstrated that species-level detection of two globally distributed thrips species (*F. occidentalis* and *E. americanus*) and two whitefly species (*B. tabaci* and *T. vaporariorum*) is achievable using high-resolution (pixel size: 5 *µm*) RGB images of yellow sticky traps and various YOLO11 and YOLO-NAS detection model versions. Although the model performance increased with higher model complexity, even the smallest studied model version (YOLO11n) resulted in acceptable macro-averaged and classwise performance scores. Considering the arbitrarily defined minimum macro-averaged practical feasibility threshold (IoU ≥ 50%) of 70% for greenhouse horticultural applications, the minimum required pixel resolution allowing species-level detection was found to be 80 *µm* for both the YOLO11n and YOLO11x models. This image resolution should, in theory, allow the use of various recent smartphones, action cameras, or even low-budget, standalone camera modules, while still requiring only a single image per sticky trap at feasible working distances. Combined with the low complexity and decent performance of the YOLO11n model, this really paves the way towards feasible, real-time, automated species-level monitoring of (yellow) sticky traps in greenhouse horticulture. Future research should further validate the feasibility/adoption in commercial IPM programs and expand this technology to more pest species, sticky trap types, and ambient light conditions.

## Data Availability

All relevant data supporting the conclusions of this article will be made available by the authors at the end of the project (September 2026) via the following Zenodo DOI: 10.5281/zenodo.15574404.

## Life Science Identifiers (LSIDs) in ZOOBANK

- ***F. occidentalis***: urn:lsid:zoobank.org:act:2EA5A102-FC8B-427F-B574-8D69155C1D2F
- ***E. americanus***: urn:lsid:zoobank.org:act:5BE86D34-19DD-4321-8AAD-E53BFAA7EE34
- ***B. tabaci***: urn:lsid:zoobank.org:act:30E31918-A380-44F9-9D97-708E74556A9D
- ***T. vaporariorum: /***
