# Supplementary material for: Species-level detection of thrips and whiteflies on yellow sticky traps using YOLO-based deep learning detection models
Source: Front Plant Sci. 2025 Nov 18;16:1668795. doi: 10.3389/fpls.2025.1668795 (PMC12669111; doi:10.3389/fpls.2025.1668795)
Supplement: Supplementary file 1 [file DataSheet1.pdf]

Supplementary material

1 ADDITIONAL FIGURES

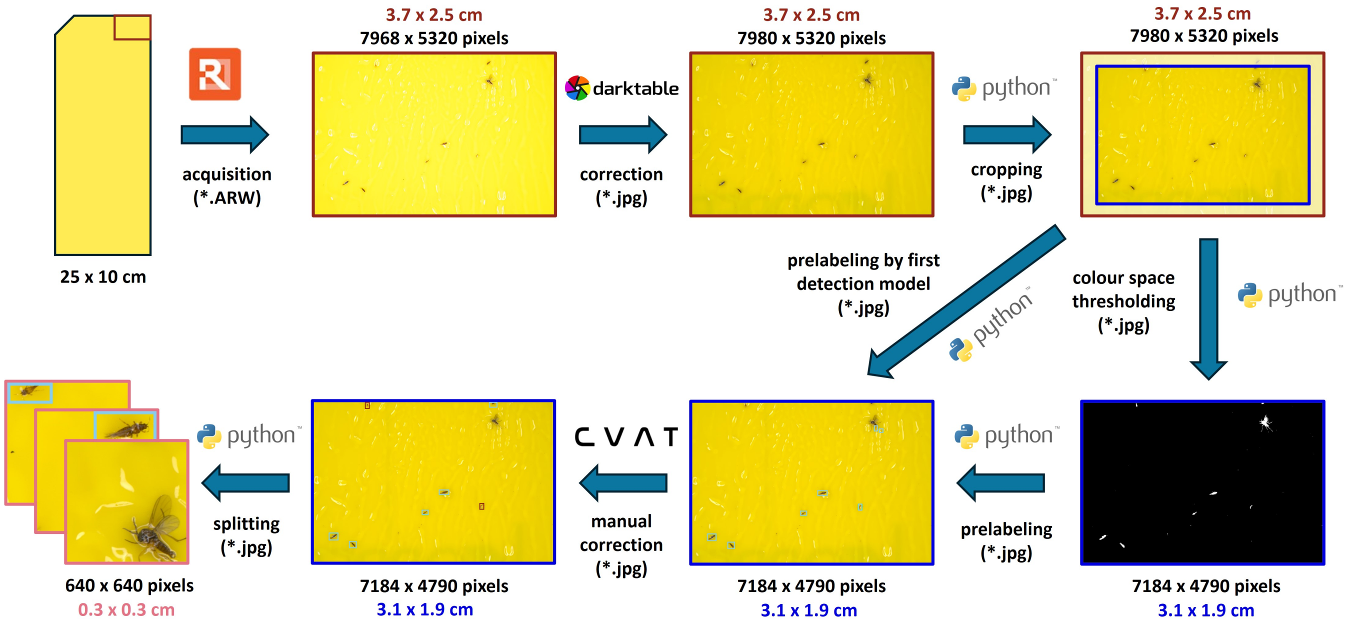

Figure S1. Overview of the applied image processing and labelling pipeline. The original high-resolution images (7968 x 5320 pixels | pixel size: 5 μm | *.ARW format) were acquired using an automated acquisition setup and the Sony Imaging Edge Desktop - Remote software. Subsequently, all images were corrected for white balance and lens distortions in Darktable and were locally stored (*.jpg format). The corrected images were later cropped to 90% of the original region of interest (Python) to avoid any potential overlap between the training and test datasets. Next, all potential present insects were prelabelled in Python using colour space conversion and thresholding or, in a later stage, a first version of the detection model. Subsequently, all labels were manually corrected using the browser version of CVAT. Finally, these images were split into smaller image patches using Python, matching the input size and file type of the studied detection models (640 x 640 pixels | *.jpg format).

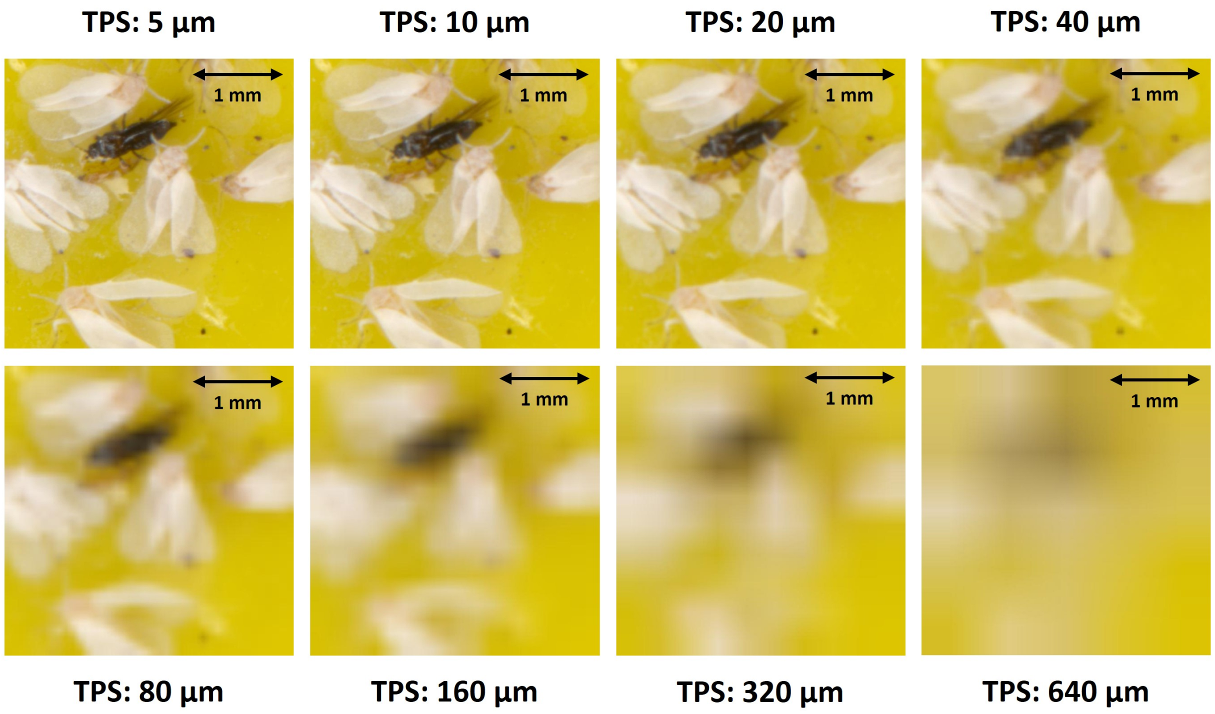

Figure S2. Exemplary image patches and corresponding theoretical pixel size (TPS) for each of the obtained reduced-resolution datasets. These datasets were obtained by resizing the original high-resolution image patch (640 x 640 pixels - pixel size: 5 μm) to a smaller dimension (factor $1/2n \mid n \in \mathbb{N}$) using bilinear interpolation, followed by resizing it back to the original image patch dimension (640 x 640 pixels), also using bilinear interpolation.

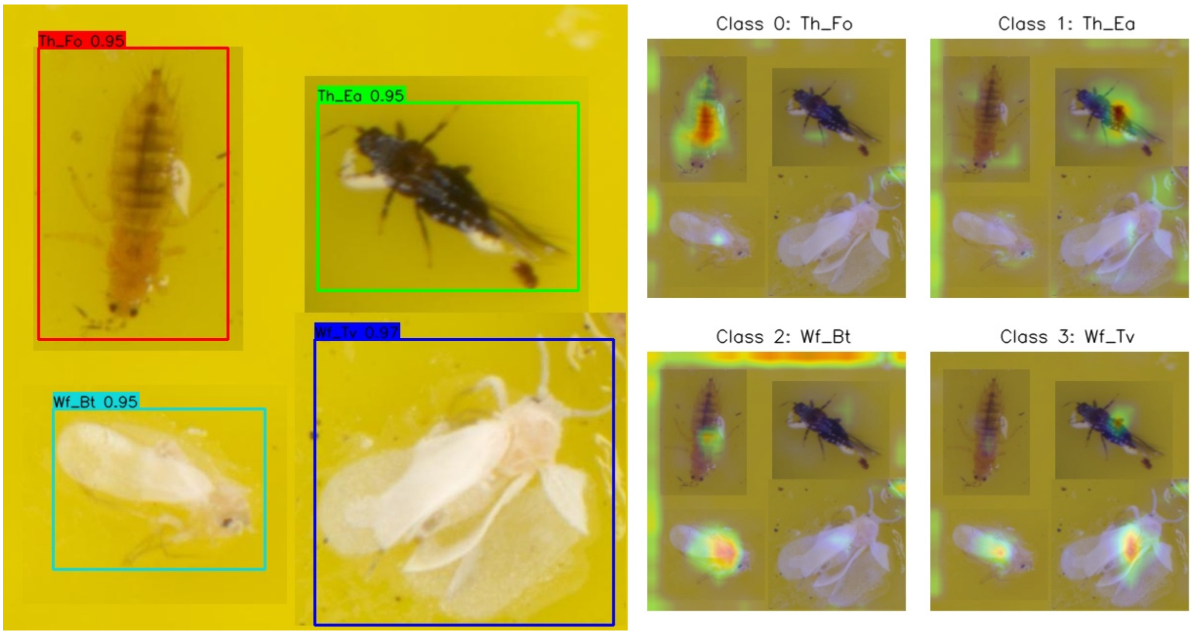

Figure S3. Detection results and classwise Grad-CAMs of the last C3k2 block in the high-resolution (pixel size: 5 μm) YOLO11n model. The mosaic was created using images originating from the external test dataset. Hereby the following label abbreviations were used: Th_Fo: *F. occidentalis* (thrips); Th_Ea: *E. americanus* (thrips); Wf_Bt: *B. tabaci* (whitefly); Wf_Tv: *T. vaporariorum* (whitefly).

2 DATASETS, SCRIPTS AND MODELS

All relevant datasets, Python scripts and trained models that were used/obtained in this study will be made available at the end of the project (September 2026) via the following Zenodo DOI: 10.5281/zenodo.15574404.

3 HIGH-RESOLUTION DATASET MODELS

3.1 model hyperparameters

3.1.1 training hyperparameters

Table S1. Specified hyperparameters during the high-resolution (pixel size: 5 μm) YOLO11 model training process in Python (ultralytics v8.3.58).

model version	YOLO11n	YOLO11s	YOLO11m	YOLO11l	YOLO11x
model	"yolo11n.pt"	"yolo11s.pt"	"yolo11m.pt"	"yolo11l.pt"	"yolo11x.pt"
task	"detect"	"detect"	"detect"	"detect"	"detect"
stop epoch	118	150	150	150	75
epochs	250	150	150	150	150
patience	100	100	100	50	15
batch	32	32	32	16	16
imgsz	640	640	640	640	640
device	0	0	0	0	0
workers	32	64	64	64	64
pretrained	True	True	True	True	True
classes	[0, 1, 2, 3]	[0, 1, 2, 3]	[0, 1, 2, 3]	[0, 1, 2, 3]	[0, 1, 2, 3]
optimizer	"Adam"	"Adam"	"Adam"	"Adam"	"Adam"
seed	0	0	0	0	0
cos_lr	True	True	True	True	True
amp	True	True	True	True	True
fraction	1	1	1	1	1
val	True	True	True	True	True
augment	True	True	True	True	True
plots	True	True	True	True	True
lr0	5.0E-05	1.0E-05	5.0E-06	5.0E-06	2.5E-06
lrf	1.0E-02	1.0E-02	1.0E-02	1.0E-02	1.0E-02
weight_decay	1.0E-04	1.0E-04	1.0E-04	1.0E-04	1.0E-04
warmup_epochs	5	5	5	5	5
warmup_bias_lr	1.0E-08	1.0E-08	1.0E-08	1.0E-08	1.0E-08

Table S2. Specified hyperparameters during the high-resolution (pixel size: 5 μm) YOLO-NAS model training process in Python (supergradients v3.6.1).

**model version**	**YOLO-NAS-S**	**YOLO-NAS-M**	**YOLO-NAS-L**
model_name	"yolo_nas_s"	"yolo_nas_m"	"yolo_nas_l"
num_classes	4	4	4
pretrained_weights	"coco"	"coco"	"coco"
device	"cuda"	"cuda"	"cuda"
stop epoch	250	150	150
max_epochs	250	150	150
batch_size	32	32	32
num_worker	32	64	32
silent_mode	False	False	False
average_best_models	True	True	True
warmup_mode	"LinearEpochLRWarmup"	"LinearEpochLRWarmup"	"LinearEpochLRWarmup"
warmup_initial_lr	1.0E-08	1.0E-07	1.0E-07
lr_warmup_epochs	5	5	5
initial_lr	5.0E-05	5.0E-05	5.0E-05
seed	42	42	42
lr_mode	"cosine"	"cosine"	"cosine"
cosine_final_lr_ratio	1.0E-02	1.0E-02	1.0E-02
optimizer	"Adam"	"Adam"	"Adam"
optimizer_params			
- weight_decay	1.0E-04	1.0E-04	1.0E-04
zero_weight_decay_on_bias_and_bn	True	True	True
ema	True	True	True
ema_params			
- decay	0.9	0.9	0.9
- decay_type	"threshold"	"threshold"	"threshold"
mixed_precision	True	True	True
loss	PPYoloELoss()	PPYoloELoss()	PPYoloELoss()
- use_static_assigner	False	False	False
- num_classes	4	4	4
metric_to_watch	"mAP@0.50:0.75"	"mAP@0.50:0.75"	"mAP@0.50:0.75"

3.1.2 testing hyperparameters

Table S3. Specified hyperparameters during the high-resolution (pixel size: 5 μm) YOLO11 model testing process in Python (ultralytics v8.3.58). The overall optimal test confidence thresholds were added for both the internal (INT) and external (EXT) test datasets.

**model name**	**YOLO11n**	**YOLO11s**	**YOLO11m**	**YOLO11l**	**YOLO11x**
conf (INT I EXT)	0.58 I 0.30	0.47 I 0.20	0.46 I 0.21	0.41 I 0.53	0.43 I 0.17
iou	0.50	0.50	0.50	0.50	0.50
batch	16	16	16	16	16
imgsz	640	640	640	640	640
max_det	10	10	10	10	10
half	False	False	False	False	False
device	"cuda:0"	"cuda:0"	"cuda:0"	"cuda:0"	"cuda:0"
plots	True	True	True	True	True
split	"test"	"test"	"test"	"test"	"test"

Table S4. Specified hyperparameters during the high-resolution (pixel size: 5 μm) YOLO-NAS model testing process in Python (super-gradients v3.6.1). The overall optimal test confidence thresholds were added for both the internal (INT) and external (EXT) test datasets.

**model name**	**YOLO-NAS-S**	**YOLO-NAS-M**	**YOLO-NAS-L**
device	"cuda"	"cuda"	"cuda"
num_workers	32	64	32
batch_size	32	32	32
score_thres (INT I EXT)	IoU@50: 0.54 I 0.57<br>IoU@75: 0.54 I 0.57<br>IoU@50:95: 0.55 I 0.57	IoU@50: 0.58 I 0.63<br>IoU@75: 0.63 I 0.63<br>IoU@50:95: 0.60 I 0.63	IoU@50: 0.55 I 0.60<br>IoU@75: 0.66 I 0.68<br>IoU@50:95: 0.64 I 0.60
top_k_predictions	10	10	10
num_cls	4	4	4
iou_thres	IoU@50: 0.50<br>IoU@75: 0.75<br>IoU@50:95: (0.5, 0.95)	IoU@50: 0.50<br>IoU@75: 0.75<br>IoU@50:95: (0.5, 0.95)	IoU@50: 0.50<br>IoU@75: 0.75<br>IoU@50:95: (0.5, 0.95)
include_classwise_ap	True	True	True
class_names	['Th_Fo', 'Th_Ea',<br>'Wf_Bt', 'Wf_Tv']	['Th_Fo', 'Th_Ea',<br>'Wf_Bt', 'Wf_Tv']	['Th_Fo', 'Th_Ea',<br>'Wf_Bt', 'Wf_Tv']
calc_best_score_thresholds	True	True	True
normalize_targets	True	True	True
post_prediction_callback	PPYoloEPostPredictionCallback()	PPYoloEPostPredictionCallback()	PPYoloEPostPredictionCallback()
- score_threshold	0.10	0.10	0.10
- nms_top_k	100	100	100
- max_predictions	10	10	10
- nms_threshold	0.50	0.50	0.50
- multi_label_per_box	False	False	False

3.2 internal test dataset performance

Table S5. Detailed internal test dataset performance metrics for each YOLO11 model version, trained on the high-resolution internal dataset (pixel size: 5 μm). The test metrics were obtained using the optimal overall confidence threshold (maximum of the F1-confidence curve) at an IoU threshold of 50%. Hereby the following abbreviations were used: Th_Fo: *F. occidentalis* (thrips); Th_Ea: *E. americanus* (thrips); Wf_Bt: *B. tabaci* (whitefly); Wf_Tv: *T. vaporariorum* (whitefly).

model version		YOLO11n	YOLO11s	YOLO11m	YOLO11l	YOLO11x
model size (M parameters)		2.6	9.4	20.1	25.3	56.9
optimal overall confidence threshold (IoU: 50%)		0.58	0.47	0.46	0.41	0.43
macro-averaged	mAP@50	0.95	0.94	0.94	0.95	0.94
	mAP@75	0.86	0.86	0.88	0.87	0.86
	mAP@50:95	0.79	0.79	0.80	0.81	0.80
	F1@50	0.93	0.92	0.92	0.93	0.92
	precision@50	0.96	0.94	0.94	0.94	0.94
	recall@50	0.90	0.90	0.91	0.92	0.90
AP@50 - classwise	Th_Fo	0.95	0.94	0.96	0.95	0.94
	Th_Ea	0.97	0.97	0.96	0.97	0.97
	Wf_Bt	0.92	0.91	0.91	0.93	0.91
	Wf_Tv	0.95	0.94	0.93	0.94	0.93
AP@50:95 - classwise	Th_Fo	0.77	0.77	0.78	0.79	0.78
	Th_Ea	0.82	0.83	0.83	0.83	0.84
	Wf_Bt	0.81	0.80	0.81	0.81	0.81
	Wf_Tv	0.78	0.76	0.78	0.78	0.76
F1@50 - classwise	Th_Fo	0.93	0.92	0.95	0.94	0.92
	Th_Ea	0.95	0.95	0.95	0.96	0.95
	Wf_Bt	0.91	0.90	0.88	0.91	0.89
	Wf_Tv	0.93	0.91	0.90	0.91	0.90
precision@50 - classwise	Th_Fo	0.97	0.96	0.97	0.96	0.96
	Th_Ea	0.96	0.96	0.97	0.97	0.96
	Wf_Bt	0.96	0.96	0.93	0.94	0.96
	Wf_Tv	0.96	0.90	0.87	0.89	0.88
recall@50 - classwise	Th_Fo	0.90	0.89	0.93	0.92	0.89
	Th_Ea	0.94	0.95	0.94	0.95	0.95
	Wf_Bt	0.86	0.84	0.84	0.88	0.83
	Wf_Tv	0.91	0.92	0.93	0.94	0.93

Table S6. Detailed internal test dataset performance metrics for each YOLO-NAS model version, trained on the high-resolution internal dataset (pixel size: 5 μm). The test metrics were obtained using the optimal overall confidence threshold (maximum of the F1-confidence curve) at the desired IoU thresholds. Hereby the following abbreviations were used: Th_Fo: *F. occidentalis* (thrips); Th_Ea: *E. americanus* (thrips); Wf_Bt: *B. tabaci* (whitefly); Wf_Tv: *T. vaporariorum* (whitefly).

model version		YOLO-NAS-S	YOLO-NAS-M	YOLO-NAS-L
model size (M parameters)		19.0	51.1	66.9
optimal overall confidence threshold		IoU@50: 0.54<br>IoU@75: 0.54<br>IoU@50:95: 0.55	IoU@50: 0.58<br>IoU@75: 0.63<br>IoU@50:95: 0.60	IoU@50: 0.55<br>IoU@75: 0.66<br>IoU@50:95: 0.64
macro-averaged	mAP@50	0.94	0.94	0.94
	mAP@75	0.79	0.79	0.77
	mAP@50:95	0.71	0.71	0.70
	F1@50	0.90	0.91	0.91
	precision@50	0.91	0.92	0.91
	recall@50	0.89	0.90	0.91
AP@50 - classwise	Th_Fo	0.94	0.95	0.97
	Th_Ea	0.97	0.97	0.98
	Wf_Bt	0.92	0.92	0.91
	Wf_Tv	0.92	0.92	0.92
AP@50:95 - classwise	Th_Fo	0.70	0.70	0.69
	Th_Ea	0.75	0.76	0.75
	Wf_Bt	0.73	0.71	0.69
	Wf_Tv	0.65	0.68	0.66
F1@50 - classwise	Th_Fo	0.92	0.92	0.94
	Th_Ea	0.93	0.92	0.93
	Wf_Bt	0.85	0.88	0.85
	Wf_Tv	0.83	0.84	0.86
precision@50 - classwise	Th_Fo	0.97	0.97	0.97
	Th_Ea	0.91	0.91	0.91
	Wf_Bt	0.88	0.91	0.88
	Wf_Tv	0.80	0.84	0.82
recall@50 - classwise	Th_Fo	0.87	0.88	0.91
	Th_Ea	0.94	0.94	0.95
	Wf_Bt	0.83	0.84	0.82
	Wf_Tv	0.86	0.84	0.90

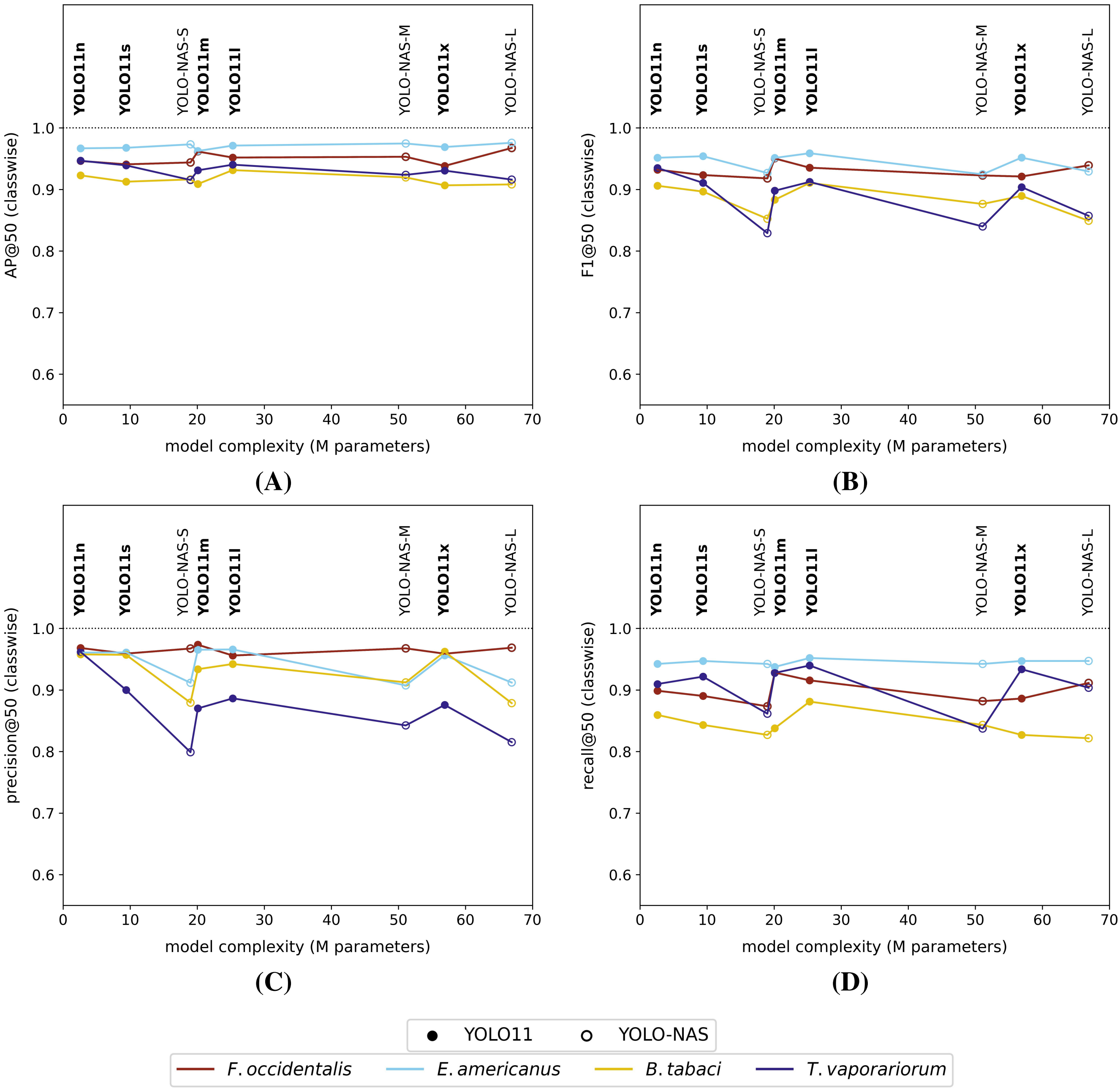

Figure S4. Classwise high-resolution (pixel size: 5 μm) internal test dataset (A) AP@50, (B) F1@50, (C) precision@50 and (D) recall@50 performance scores for each of the studied model versions, trained on the high-resolution internal dataset.

3.3 external test dataset performance

Table S7. Detailed external test dataset performance metrics for each YOLO11 model version, trained on the high-resolution internal dataset (pixel size: 5 μm). The test metrics were obtained using the optimal overall confidence threshold (maximum of the F1-confidence curve) at an IoU threshold of 50%. Hereby the following abbreviations were used: Th_Fo: *F. occidentalis* (thrips); Th_Ea: *E. americanus* (thrips); Wf_Bt: *B. tabaci* (whitefly); Wf_Tv: *T. vaporariorum* (whitefly).

model version		YOLO11n	YOLO11s	YOLO11m	YOLO11l	YOLO11x
model size (M parameters)		2.6	9.4	20.1	25.3	56.9
optimal overall confidence threshold (IoU: 50%)		0.30	0.20	0.21	0.53	0.17
macro-averaged	mAP@50	0.80	0.79	0.81	0.80	0.84
	mAP@75	0.76	0.74	0.77	0.76	0.79
	mAP@50:95	0.68	0.67	0.70	0.70	0.73
	F1@50	0.77	0.74	0.77	0.77	0.81
	precision@50	0.78	0.76	0.80	0.86	0.82
	recall@50	0.77	0.74	0.78	0.71	0.82
AP@50 - classwise	Th_Fo	0.72	0.69	0.74	0.75	0.85
	Th_Ea	0.84	0.80	0.84	0.86	0.88
	Wf_Bt	0.85	0.86	0.85	0.81	0.88
	Wf_Tv	0.78	0.82	0.83	0.79	0.77
AP@50:95 - classwise	Th_Fo	0.62	0.56	0.65	0.67	0.74
	Th_Ea	0.66	0.63	0.66	0.68	0.70
	Wf_Bt	0.75	0.75	0.76	0.72	0.79
	Wf_Tv	0.69	0.73	0.74	0.73	0.69
F1@50 - classwise	Th_Fo	0.68	0.62	0.69	0.70	0.82
	Th_Ea	0.82	0.76	0.82	0.83	0.85
	Wf_Bt	0.81	0.82	0.81	0.79	0.85
	Wf_Tv	0.77	0.75	0.77	0.76	0.71
precision@50 - classwise	Th_Fo	0.69	0.63	0.74	0.86	0.88
	Th_Ea	0.95	0.94	0.89	0.95	0.87
	Wf_Bt	0.83	0.86	0.93	0.93	0.93
	Wf_Tv	0.66	0.62	0.63	0.71	0.59
recall@50 - classwise	Th_Fo	0.67	0.61	0.65	0.59	0.78
	Th_Ea	0.72	0.63	0.76	0.74	0.83
	Wf_Bt	0.80	0.78	0.72	0.69	0.78
	Wf_Tv	0.91	0.94	0.98	0.83	0.89

Table S8. Detailed external test dataset performance metrics for each YOLO-NAS model version, trained on the high-resolution internal dataset (pixel size: 5 μm). The test metrics were obtained using the optimal overall confidence threshold (maximum of the F1-confidence curve) at the desired IoU thresholds. Hereby the following abbreviations were used: Th_Fo: *F. occidentalis* (thrips); Th_Ea: *E. americanus* (thrips); Wf_Bt: *B. tabaci* (whitefly); Wf_Tv: *T. vaporariorum* (whitefly).

**model version**		**YOLO-NAS-S**	**YOLO-NAS-M**	**YOLO-NAS-L**
model size (M parameters)		19.0	51.1	66.9
optimal overall confidence threshold		IoU@50: 0.57<br>IoU@75: 0.57<br>IoU@50:95: 0.57	IoU@50: 0.63<br>IoU@75: 0.63<br>IoU@50:95: 0.63	IoU@50: 0.60<br>IoU@75: 0.68<br>IoU@50:95: 0.60
macro-averaged	mAP@50	0.86	0.85	0.89
	mAP@75	0.81	0.80	0.81
	mAP@50:95	0.72	0.70	0.71
	F1@50	0.83	0.86	0.87
	precision@50	0.84	0.89	0.88
	recall@50	0.82	0.83	0.87
AP@50 - classwise	Th_Fo	0.93	0.91	0.95
	Th_Ea	0.95	0.93	0.95
	Wf_Bt	0.78	0.80	0.82
	Wf_Tv	0.78	0.78	0.82
AP@50:95 - classwise	Th_Fo	0.81	0.75	0.80
	Th_Ea	0.76	0.74	0.72
	Wf_Bt	0.68	0.71	0.68
	Wf_Tv	0.63	0.61	0.64
F1@50 - classwise	Th_Fo	0.85	0.89	0.83
	Th_Ea	0.90	0.90	0.94
	Wf_Bt	0.77	0.74	0.81
	Wf_Tv	0.74	0.75	0.76
precision@50 - classwise	Th_Fo	0.88	0.94	0.83
	Th_Ea	0.92	0.92	1.00
	Wf_Bt	0.74	0.72	0.77
	Wf_Tv	0.72	0.82	0.76
recall@50 - classwise	Th_Fo	0.81	0.85	0.83
	Th_Ea	0.87	0.87	0.89
	Wf_Bt	0.80	0.76	0.85
	Wf_Tv	0.76	0.69	0.76

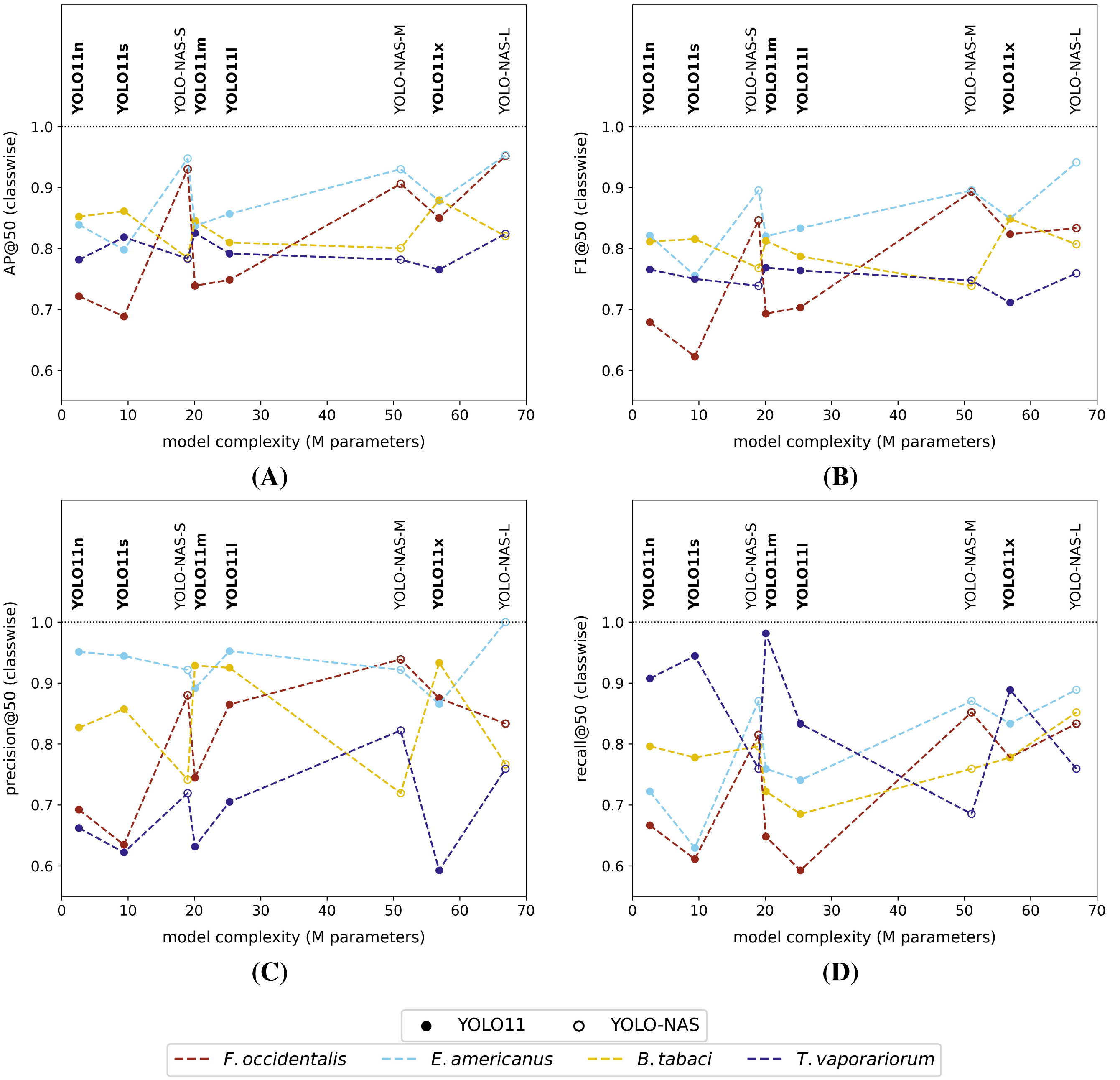

Figure S5. Classwise high-resolution (pixel size: 5 μm) external test dataset (A) AP@50, (B) F1@50, (C) precision@50 and (D) recall@50 performance scores for each of the studied model versions, trained on the high-resolution internal dataset.

4 REDUCED-RESOLUTION DATASET MODELS

4.1 model hyperparameters

4.1.1 training hyperparameters

Table S9. Specified hyperparameters during the reduced-resolution (theoretical pixel size: 5 μm - 640 μm) YOLO11n model training process in Python (ultralytics v8.3.58).

theoretical pixel size (µm)	5	10	20	40	80	160	320	640
model	"yolo11n.pt"	"yolo11n.pt"	"yolo11n.pt"	"yolo11n.pt"	"yolo11n.pt"	"yolo11n.pt"	"yolo11n.pt"	"yolo11n.pt"
task	"detect"	"detect"	"detect"	"detect"	"detect"	"detect"	"detect"	"detect"
stop epoch	150	60	137	136	127	132	120	130
epochs	150	150	150	150	150	150	150	150
patience	50	25	20	20	20	100	100	100
batch	32	32	32	32	32	32	32	32
imgsz	640	640	640	640	640	640	640	640
device	0	"cpu"	"cpu"	"cpu"	"cpu"	0	0	0
workers	64	64	64	64	64	64	64	64
pretrained	True	True	True	True	True	True	True	True
classes	[0, 1, 2, 3]	[0, 1, 2, 3]	[0, 1, 2, 3]	[0, 1, 2, 3]	[0, 1, 2, 3]	[0, 1, 2, 3]	[0, 1, 2, 3]	[0, 1, 2, 3]
optimizer	"Adam"	"Adam"	"Adam"	"Adam"	"Adam"	"Adam"	"Adam"	"Adam"
seed	0	0	0	0	0	0	0	0
cos_lr	True	True	True	True	True	True	True	True
amp	True	True	True	True	True	True	True	True
fraction	1	1	1	1	1	1	1	1
val	True	True	True	True	True	True	True	True
augment	True	True	True	True	True	True	True	True
plots	True	True	True	True	True	True	True	True
lr0	1.0E-04	1.0E-04	1.0E-04	1.0E-04	1.0E-04	1.0E-04	1.0E-04	1.0E-04
lrf	0.01	0.01	0.01	0.01	0.01	0.01	0.01	0.01
weight_decay	1.0E-04	1.0E-04	1.0E-04	1.0E-04	1.0E-04	1.0E-04	1.0E-04	1.0E-04
warmup_epochs	5	5	5	5	5	5	5	5
warmup_bias_lr	1.0E-08	1.0E-08	1.0E-08	1.0E-08	1.0E-08	1.0E-08	1.0E-08	1.0E-08

Table S10. Specified hyperparameters during the reduced-resolution (theoretical pixel size: 5 μm - 640 μm) YOLO11x model training process in Python (ultralytics v8.3.58).

theoretical pixel size (µm)	5	10	20	40	80	160	320	640
model	"yolo11x.pt"	"yolo11x.pt"	"yolo11x.pt"	"yolo11x.pt"	"yolo11x.pt"	"yolo11x.pt"	"yolo11x.pt"	"yolo11x.pt"
task	"detect"	"detect"	"detect"	"detect"	"detect"	"detect"	"detect"	"detect"
stop epoch	136	122	134	122	141	130	130	130
epochs	150	150	150	150	150	150	150	150
patience	20	20	20	20	20	100	100	100
batch	16	16	32	16	16	16	16	16
imgsz	640	640	640	640	640	640	640	640
device	0	0	0	0	0	0	0	0
workers	64	64	64	64	64	64	64	64
pretrained	True	True	True	True	True	True	True	True
classes	[0, 1, 2, 3]	[0, 1, 2, 3]	[0, 1, 2, 3]	[0, 1, 2, 3]	[0, 1, 2, 3]	[0, 1, 2, 3]	[0, 1, 2, 3]	[0, 1, 2, 3]
optimizer	"Adam"	"Adam"	"Adam"	"Adam"	"Adam"	"Adam"	"Adam"	"Adam"
seed	0	0	0	0	0	0	0	0
cos_lr	True	True	True	True	True	True	True	True
amp	True	True	True	True	True	True	True	True
fraction	1	1	1	1	1	1	1	1
val	True	True	True	True	True	True	True	True
augment	True	True	True	True	True	True	True	True
plots	True	True	True	True	True	True	True	True
lr0	2.5E-06	2.5E-06	2.5E-06	2.5E-06	2.5E-06	2.5E-06	2.5E-06	2.5E-06
lrf	1.0E-02	1.0E-02	1.0E-02	1.0E-02	1.0E-02	1.0E-02	1.0E-02	1.0E-02
weight_decay	1.0E-04	1.0E-04	1.0E-04	1.0E-04	1.0E-04	1.0E-04	1.0E-04	1.0E-04
warmup_epochs	5	5	5	5	5	5	5	5
warmup_bias_lr	1.0E-08	1.0E-08	1.0E-08	1.0E-08	1.0E-08	1.0E-08	1.0E-08	1.0E-08

4.1.2 testing hyperparameters

Table S11. Specified hyperparameters during the reduced-resolution (theoretical pixel size: 5 μm - 640 μm) YOLO11n model testing process in Python (ultralytics v8.3.58). The overall optimal test confidence thresholds were added for both the internal (INT) and external (EXT) test datasets.

theoretical pixel size (µm)	5	10	20	40	80	160	320	640
conf (INT I EXT)	0.54 I 0.31	0.45 I 0.35	0.44 I 0.49	0.48 I 0.39	0.39 I 0.38	0.30 I 0.23	0.26 I 0.39	0.14 I 0.14
iou	0.50	0.50	0.50	0.50	0.50	0.50	0.50	0.50
batch	16	16	16	16	16	16	16	16
imgsz	640	640	640	640	640	640	640	640
max_det	10	10	10	10	10	10	10	10
half	False	False	False	False	False	False	False	False
device	"cuda:0"	"cuda:0"	"cuda:0"	"cuda:0"	"cuda:0"	"cuda:0"	"cuda:0"	"cuda:0"
plots	True	True	True	True	True	True	True	True
split	"test"	"test"	"test"	"test"	"test"	"test"	"test"	"test"

Table S12. Specified hyperparameters during the reduced-resolution (theoretical pixel size: 5 μm - 640 μm) YOLO11x model testing process in Python (ultralytics v8.3.58). The overall optimal test confidence thresholds were added for both the internal (INT) and external (EXT) test datasets.

theoretical pixel size (µm)	5	10	20	40	80	160	320	640
conf (INT I EXT)	0.43 I 0.38	0.37 I 0.17	0.30 I 0.19	0.41 I 0.29	0.4 I 0.24	0.43 I 0.11	0.14 I 0.09	0.14 I 0.10
iou	0.50	0.50	0.50	0.50	0.50	0.50	0.50	0.50
batch	16	16	16	16	16	16	16	16
imgsz	640	640	640	640	640	640	640	640
max_det	10	10	10	10	10	10	10	10
half	False	False	False	False	False	False	False	False
device	"cuda:0"	"cuda:0"	"cuda:0"	"cuda:0"	"cuda:0"	"cuda:0"	"cuda:0"	"cuda:0"
plots	True	True	True	True	True	True	True	True
split	"test"	"test"	"test"	"test"	"test"	"test"	"test"	"test"

4.2 internal test dataset performance

Table S13. Detailed internal test dataset performance metrics for each YOLO11n model version, trained on the corresponding reduced-resolution internal datasets (theoretical pixel size: 5 μm - 640 μm). The test metrics were obtained using the optimal overall confidence threshold (maximum of the F1-confidence curve) at an IoU threshold of 50%. Hereby the following abbreviations were used: Th_Fo: *F. occidentalis* (thrips); Th_Ea: *E. americanus* (thrips); Wf_Bt: *B. tabaci* (whitefly); Wf_Tv: *T. vaporariorum* (whitefly).

**theoretical pixel size (µm)**		**5**	**10**	**20**	**40**	**80**	**160**	**320**	**640**
optimal overall confidence threshold (IoU: 50%)		0.54	0.45	0.44	0.48	0.39	0.30	0.26	0.14
macro-averaged	mAP@50	0.95	0.93	0.95	0.94	0.91	0.87	0.77	0.60
	mAP@75	0.87	0.85	0.85	0.85	0.77	0.61	0.41	0.16
	mAP@50:95	0.79	0.77	0.77	0.75	0.66	0.55	0.42	0.25
	F1@50	0.92	0.91	0.91	0.92	0.87	0.83	0.74	0.60
	precision@50	0.94	0.94	0.92	0.93	0.90	0.85	0.74	0.60
	recall@50	0.91	0.88	0.91	0.91	0.85	0.82	0.74	0.60
AP@50 - classwise	Th_Fo	0.95	0.95	0.95	0.95	0.91	0.86	0.76	0.54
	Th_Ea	0.97	0.96	0.96	0.97	0.96	0.94	0.86	0.63
	Wf_Bt	0.93	0.90	0.93	0.92	0.88	0.85	0.76	0.70
	Wf_Tv	0.93	0.91	0.94	0.93	0.88	0.83	0.69	0.52
AP@50:95 - classwise	Th_Fo	0.75	0.76	0.76	0.74	0.66	0.52	0.40	0.22
	Th_Ea	0.82	0.80	0.81	0.79	0.71	0.62	0.45	0.22
	Wf_Bt	0.82	0.78	0.79	0.75	0.67	0.56	0.44	0.34
	Wf_Tv	0.77	0.74	0.74	0.70	0.61	0.50	0.37	0.22
F1@50 - classwise	Th_Fo	0.93	0.93	0.92	0.93	0.88	0.83	0.72	0.52
	Th_Ea	0.95	0.94	0.94	0.96	0.93	0.89	0.82	0.65
	Wf_Bt	0.91	0.88	0.91	0.91	0.86	0.80	0.75	0.67
	Wf_Tv	0.91	0.88	0.88	0.88	0.83	0.81	0.67	0.54
precision@50 - classwise	Th_Fo	0.95	0.95	0.93	0.92	0.90	0.89	0.79	0.61
	Th_Ea	0.95	0.96	0.94	0.98	0.92	0.84	0.75	0.60
	Wf_Bt	0.93	0.96	0.95	0.96	0.93	0.80	0.77	0.63
	Wf_Tv	0.93	0.87	0.84	0.86	0.85	0.87	0.66	0.55
recall@50 - classwise	Th_Fo	0.90	0.90	0.92	0.93	0.86	0.78	0.65	0.46
	Th_Ea	0.95	0.93	0.94	0.95	0.94	0.94	0.89	0.71
	Wf_Bt	0.89	0.82	0.88	0.85	0.79	0.81	0.73	0.72
	Wf_Tv	0.90	0.88	0.92	0.90	0.81	0.75	0.67	0.52

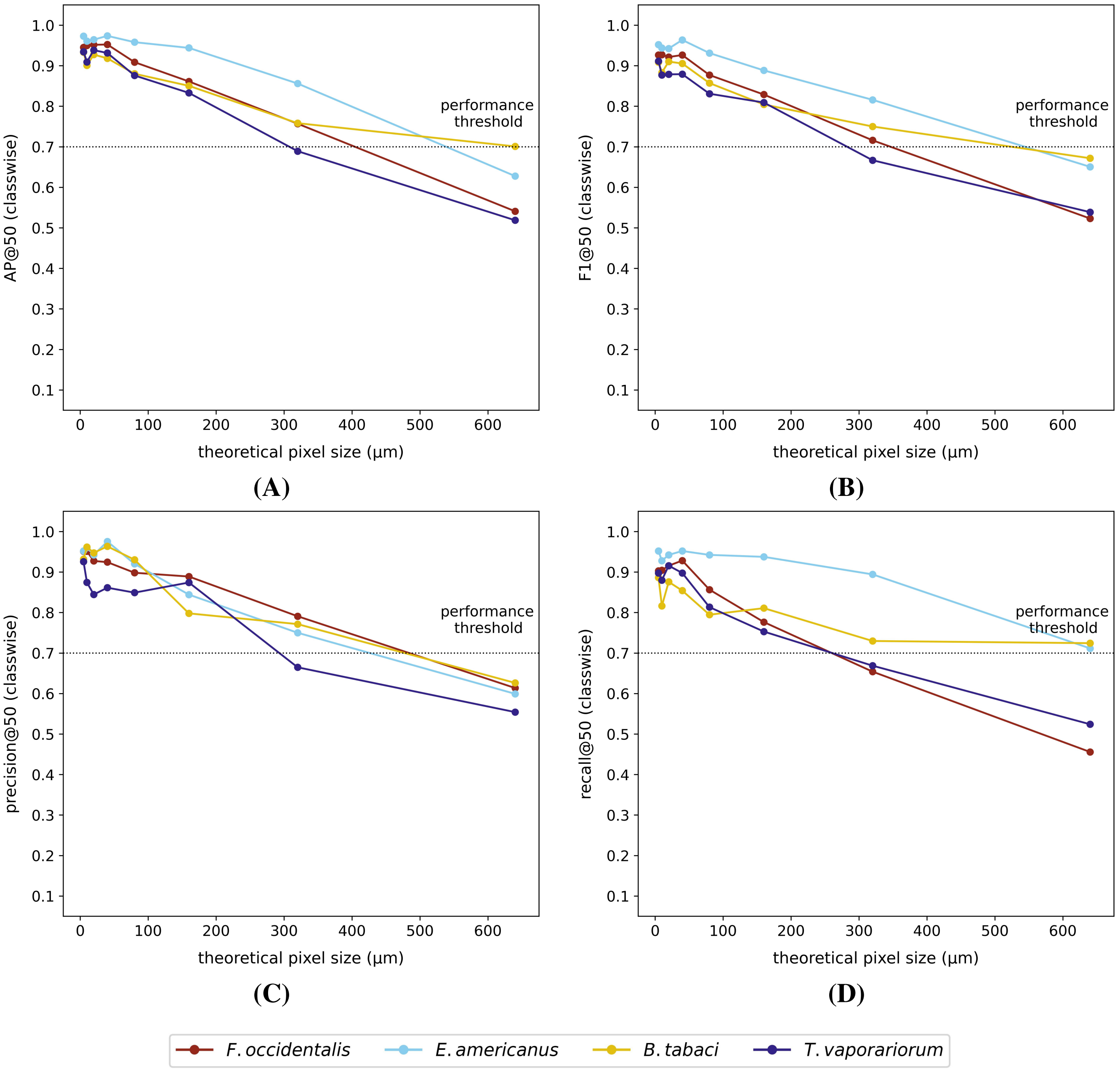

Figure S6. Classwise internal test dataset (A) AP@50, (B) F1@50, (C) precision@50 and (D) recall@50 performance scores for each of the YOLO11n model versions, trained on the corresponding reduced-resolution internal datasets (theoretical pixel size: 5 μm - 640 μm). The black dotted line represents the arbitrarily defined minimum required practical feasibility threshold of 70% for greenhouse horticultural applications.

Table S14. Detailed internal test dataset performance metrics for each YOLO11x model version, trained on the corresponding reduced-resolution internal datasets (theoretical pixel size: 5 μm - 640 μm). The test metrics were obtained using the optimal overall confidence threshold (maximum of the F1-confidence curve) at an IoU threshold of 50%. Hereby the following abbreviations were used: Th_Fo: *F. occidentalis* (thrips); Th_Ea: *E. americanus* (thrips); Wf_Bt: *B. tabaci* (whitefly); Wf_Tv: *T. vaporariorum* (whitefly).

**theoretical pixel size (µm)**		**5**	**10**	**20**	**40**	**80**	**160**	**320**	**640**
optimal overall confidence threshold (IoU: 50%)		0.43	0.37	0.30	0.41	0.40	0.43	0.14	0.14
macro-averaged	mAP@50	0.95	0.95	0.95	0.93	0.90	0.84	0.75	0.64
	mAP@75	0.88	0.87	0.86	0.84	0.76	0.61	0.39	0.20
	mAP@50:95	0.81	0.80	0.78	0.75	0.66	0.54	0.41	0.28
	F1@50	0.93	0.93	0.92	0.91	0.87	0.82	0.72	0.62
	precision@50	0.94	0.93	0.93	0.93	0.89	0.87	0.74	0.66
	recall@50	0.91	0.92	0.92	0.89	0.86	0.77	0.73	0.60
AP@50 - classwise	Th_Fo	0.95	0.95	0.95	0.94	0.92	0.86	0.73	0.59
	Th_Ea	0.97	0.97	0.97	0.96	0.95	0.91	0.82	0.69
	Wf_Bt	0.92	0.94	0.93	0.92	0.86	0.84	0.79	0.72
	Wf_Tv	0.94	0.94	0.93	0.92	0.85	0.75	0.68	0.56
AP@50:95 - classwise	Th_Fo	0.79	0.79	0.77	0.75	0.66	0.52	0.40	0.27
	Th_Ea	0.83	0.82	0.81	0.78	0.72	0.64	0.41	0.25
	Wf_Bt	0.83	0.82	0.79	0.78	0.66	0.53	0.45	0.38
	Wf_Tv	0.80	0.78	0.76	0.70	0.58	0.48	0.37	0.24
F1@50 - classwise	Th_Fo	0.94	0.93	0.93	0.91	0.89	0.83	0.70	0.54
	Th_Ea	0.96	0.95	0.95	0.95	0.93	0.90	0.79	0.69
	Wf_Bt	0.90	0.92	0.91	0.90	0.85	0.81	0.77	0.68
	Wf_Tv	0.91	0.90	0.91	0.89	0.81	0.74	0.63	0.58
precision@50 - classwise	Th_Fo	0.98	0.96	0.93	0.94	0.91	0.87	0.67	0.71
	Th_Ea	0.97	0.96	0.95	0.97	0.93	0.95	0.91	0.72
	Wf_Bt	0.95	0.96	0.95	0.95	0.94	0.92	0.81	0.69
	Wf_Tv	0.89	0.86	0.88	0.89	0.78	0.74	0.54	0.52
recall@50 - classwise	Th_Fo	0.90	0.91	0.92	0.89	0.87	0.79	0.73	0.44
	Th_Ea	0.95	0.95	0.95	0.93	0.93	0.85	0.70	0.66
	Wf_Bt	0.86	0.89	0.88	0.85	0.78	0.72	0.72	0.66
	Wf_Tv	0.93	0.94	0.94	0.89	0.84	0.73	0.77	0.64

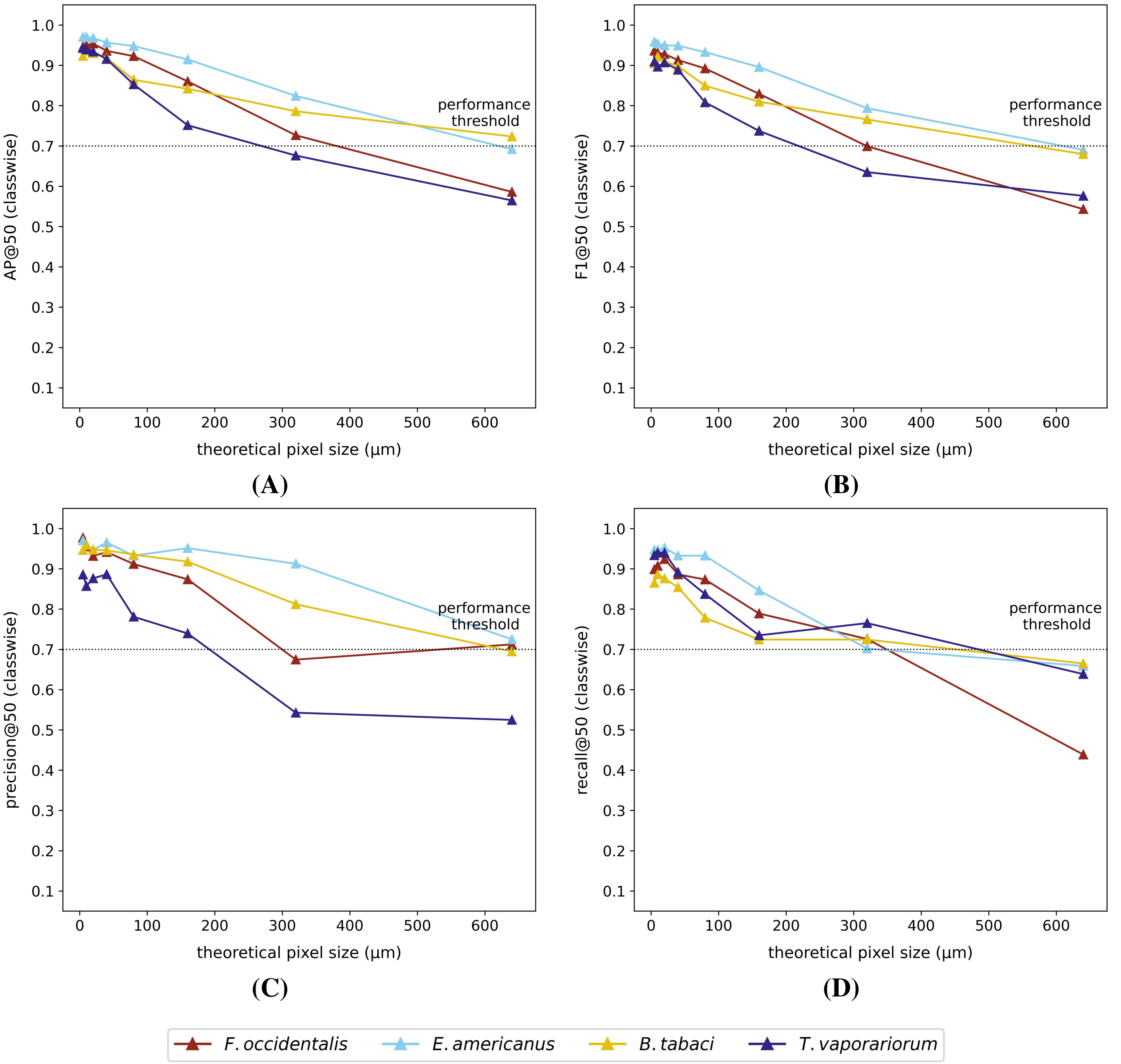

Figure S7. Classwise internal test dataset (A) AP@50, (B) F1@50, (C) precision@50 and (D) recall@50 performance scores for each of the YOLO11x model versions, trained on the corresponding reduced-resolution internal datasets (theoretical pixel size: 5 μm - 640 μm). The black dotted line represents the arbitrarily defined minimum required practical feasibility threshold of 70% for greenhouse horticultural applications.

4.3 external text dataset performance

Table S15. Detailed external test dataset performance metrics for each YOLO11n model version, trained on the corresponding reduced-resolution internal datasets (theoretical pixel size: 5 μm - 640 μm). The test metrics were obtained using the optimal overall confidence threshold (maximum of the F1-confidence curve) at an IoU threshold of 50%. Hereby the following abbreviations were used: Th_Fo: *F. occidentalis* (thrips); Th_Ea: *E. americanus* (thrips); Wf_Bt: *B. tabaci* (whitefly); Wf_Tv: *T. vaporariorum* (whitefly).

theoretical pixel size (µm)		5	10	20	40	80	160	320	640
optimal overall confidence threshold (IoU: 50%)		0.31	0.35	0.49	0.39	0.38	0.23	0.39	0.14
macro-averaged	mAP@50	0.85	0.77	0.84	0.84	0.82	0.69	0.62	0.55
	mAP@75	0.79	0.73	0.81	0.79	0.72	0.50	0.38	0.17
	mAP@50:95	0.73	0.66	0.72	0.68	0.60	0.44	0.36	0.26
	F1@50	0.82	0.74	0.82	0.80	0.77	0.65	0.62	0.54
	precision@50	0.84	0.81	0.88	0.84	0.82	0.68	0.68	0.51
	recall@50	0.82	0.71	0.78	0.79	0.75	0.68	0.60	0.59
AP@50 - classwise	Th_Fo	0.80	0.71	0.76	0.75	0.74	0.41	0.29	0.24
	Th_Ea	0.90	0.76	0.83	0.83	0.85	0.59	0.67	0.54
	Wf_Bt	0.86	0.83	0.89	0.86	0.81	0.88	0.75	0.73
	Wf_Tv	0.83	0.77	0.88	0.89	0.87	0.86	0.77	0.72
AP@50:95 - classwise	Th_Fo	0.69	0.62	0.65	0.61	0.55	0.27	0.17	0.12
	Th_Ea	0.71	0.61	0.71	0.67	0.58	0.35	0.34	0.20
	Wf_Bt	0.75	0.75	0.77	0.71	0.63	0.55	0.45	0.34
	Wf_Tv	0.76	0.67	0.76	0.74	0.65	0.58	0.47	0.37
F1@50 - classwise	Th_Fo	0.78	0.68	0.74	0.74	0.71	0.45	0.38	0.29
	Th_Ea	0.87	0.71	0.81	0.80	0.82	0.53	0.60	0.57
	Wf_Bt	0.82	0.80	0.87	0.84	0.77	0.81	0.77	0.63
	Wf_Tv	0.81	0.77	0.84	0.82	0.78	0.83	0.74	0.67
precision@50 - classwise	Th_Fo	0.86	0.66	0.88	0.76	0.80	0.40	0.39	0.29
	Th_Ea	0.88	0.97	0.95	0.91	0.95	0.81	0.86	0.60
	Wf_Bt	0.93	0.95	0.94	1.00	0.84	0.77	0.79	0.53
	Wf_Tv	0.69	0.67	0.76	0.70	0.69	0.73	0.69	0.60
recall@50 - classwise	Th_Fo	0.70	0.70	0.65	0.72	0.65	0.52	0.37	0.28
	Th_Ea	0.85	0.56	0.70	0.72	0.72	0.39	0.46	0.54
	Wf_Bt	0.74	0.69	0.81	0.72	0.70	0.85	0.76	0.78
	Wf_Tv	0.98	0.91	0.94	0.98	0.91	0.96	0.80	0.76

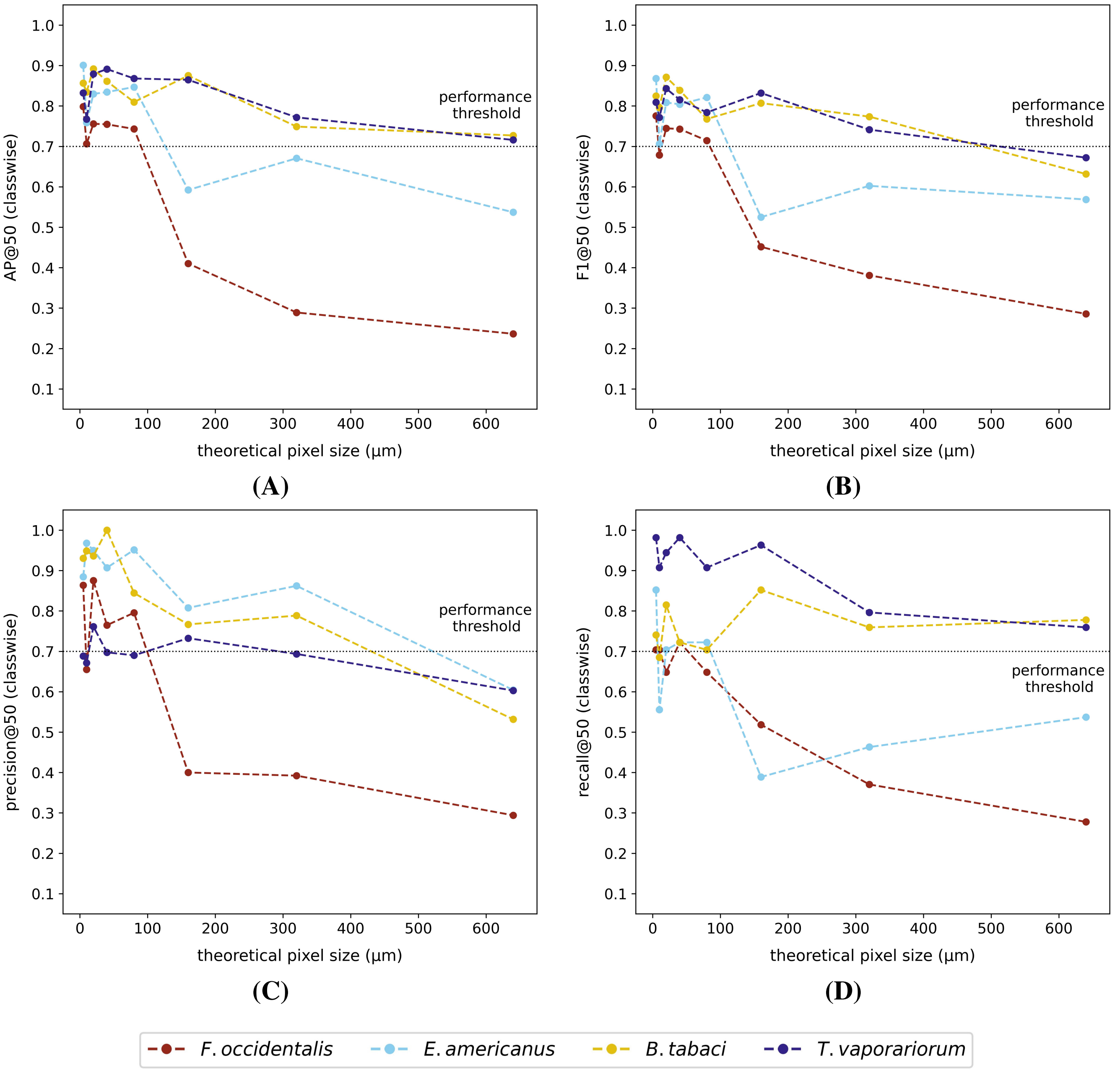

Figure S8. Classwise external test dataset (A) AP@50, (B) F1@50, (C) precision@50 and (D) recall@50 performance scores for each of the YOLO11n model versions, trained on the corresponding reduced-resolution internal datasets (theoretical pixel size: 5 μm - 640 μm). The black dotted line represents the arbitrarily defined minimum required practical feasibility threshold of 70% for greenhouse horticultural applications.

Table S16. Detailed external test dataset performance metrics for each YOLO11x model version, trained on the corresponding reduced-resolution internal datasets (theoretical pixel size: 5 μm - 640 μm). The test metrics were obtained using the optimal overall confidence threshold (maximum of the F1-confidence curve) at an IoU threshold of 50%. Hereby the following abbreviations were used: Th_Fo: *F. occidentalis* (thrips); Th_Ea: *E. americanus* (thrips); Wf_Bt: *B. tabaci* (whitefly); Wf_Tv: *T. vaporariorum* (whitefly).

**theoretical pixel size (µm)**		**5**	**10**	**20**	**40**	**80**	**160**	**320**	**640**
optimal overall confidence threshold (IoU: 50%)		0.38	0.17	0.19	0.29	0.24	0.11	0.09	0.10
macro-averaged	mAP@50	0.80	0.85	0.84	0.83	0.80	0.69	0.61	0.54
	mAP@75	0.76	0.80	0.82	0.78	0.68	0.47	0.38	0.20
	mAP@50:95	0.70	0.74	0.73	0.68	0.58	0.43	0.36	0.26
	F1@50	0.78	0.81	0.80	0.79	0.75	0.59	0.50	0.51
	precision@50	0.85	0.81	0.84	0.84	0.79	0.62	0.56	0.54
	recall@50	0.74	0.82	0.79	0.76	0.75	0.70	0.60	0.54
AP@50 - classwise	Th_Fo	0.77	0.86	0.85	0.82	0.73	0.47	0.40	0.22
	Th_Ea	0.80	0.86	0.84	0.80	0.73	0.56	0.44	0.48
	Wf_Bt	0.85	0.86	0.85	0.86	0.89	0.87	0.82	0.79
	Wf_Tv	0.79	0.83	0.82	0.83	0.85	0.84	0.77	0.69
AP@50:95 - classwise	Th_Fo	0.67	0.74	0.75	0.70	0.53	0.28	0.21	0.11
	Th_Ea	0.64	0.71	0.68	0.63	0.51	0.28	0.23	0.18
	Wf_Bt	0.76	0.76	0.76	0.71	0.67	0.58	0.49	0.39
	Wf_Tv	0.73	0.74	0.72	0.66	0.62	0.59	0.49	0.36
F1@50 - classwise	Th_Fo	0.74	0.82	0.82	0.82	0.71	0.52	0.44	0.26
	Th_Ea	0.80	0.84	0.83	0.77	0.68	0.44	0.25	0.43
	Wf_Bt	0.82	0.84	0.82	0.82	0.88	0.80	0.73	0.72
	Wf_Tv	0.76	0.73	0.73	0.76	0.74	0.62	0.60	0.63
precision@50 - classwise	Th_Fo	0.88	0.83	0.84	0.86	0.65	0.42	0.37	0.32
	Th_Ea	0.93	0.90	0.95	0.95	0.94	0.84	0.73	0.68
	Wf_Bt	0.93	0.91	0.95	0.89	0.98	0.75	0.69	0.68
	Wf_Tv	0.68	0.60	0.62	0.68	0.62	0.45	0.44	0.50
recall@50 - classwise	Th_Fo	0.65	0.81	0.80	0.78	0.78	0.69	0.56	0.22
	Th_Ea	0.70	0.80	0.74	0.65	0.54	0.30	0.15	0.31
	Wf_Bt	0.74	0.78	0.72	0.76	0.80	0.85	0.76	0.76
	Wf_Tv	0.87	0.91	0.89	0.87	0.91	0.96	0.94	0.85

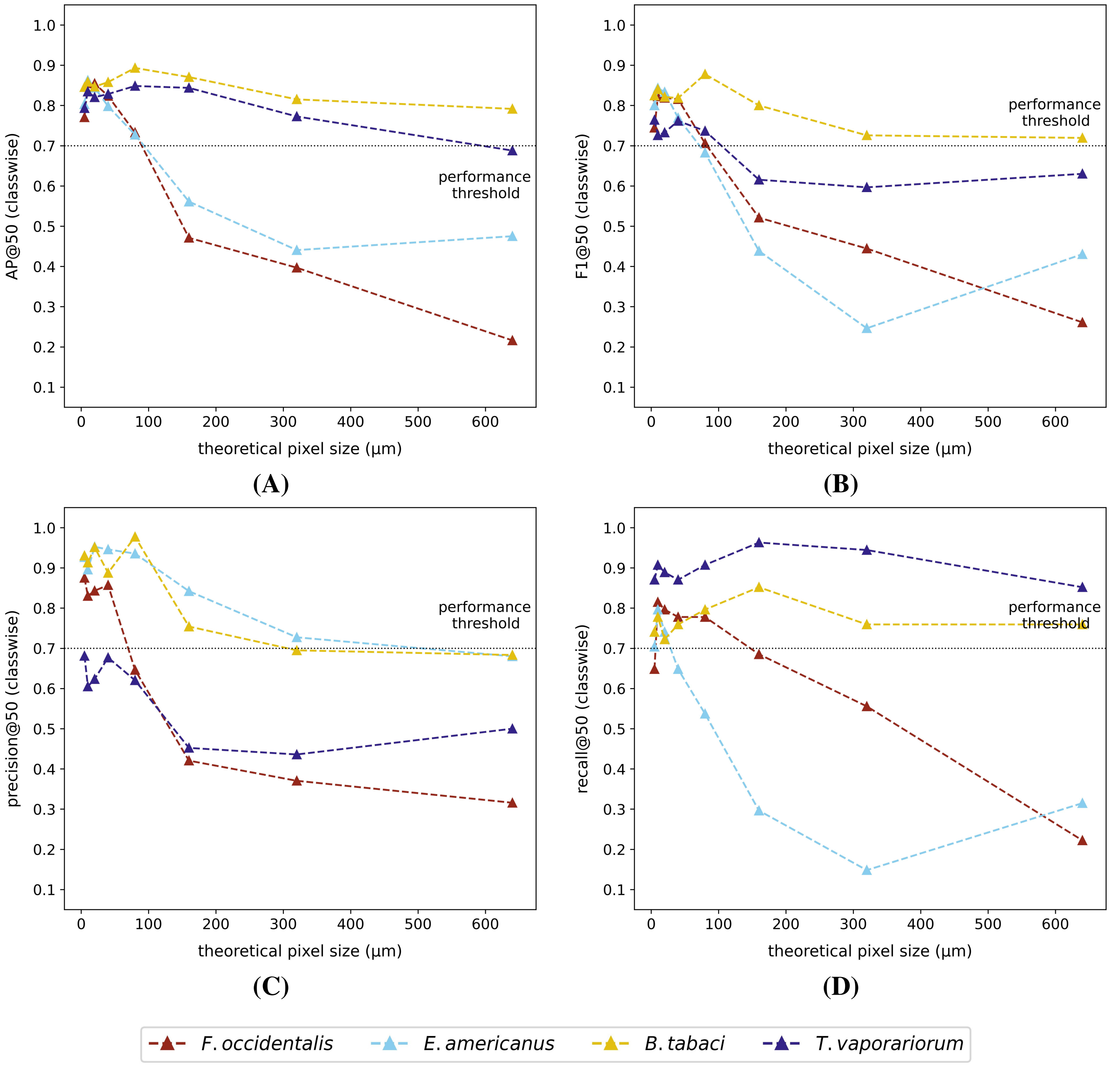

Figure S9. Classwise external test dataset (A) AP@50, (B) F1@50, (C) precision@50 and (D) recall@50 performance scores for each of the YOLO11x model versions, trained on the corresponding reduced-resolution internal datasets (theoretical pixel size: 5 μm - 640 μm). The black dotted line represents the arbitrarily defined minimum required practical feasibility threshold of 70% for greenhouse horticultural applications.

5 EQUATIONS/FORMULA

5.1 Model test performance metrics

$$precision@50 = \frac{TP}{TP + FP} \qquad [\text{IoU} \geq 50\%] \tag{S1}$$

where:

- TP: true positives or correct detections, calculated at an IoU threshold of 50%
- FP: false positives or incorrect detections, calculated at an IoU threshold of 50%

$$recall@50 = \frac{TP}{TP + FN} \qquad [\text{IoU} \geq 50\%] \tag{S2}$$

where:

- TP: true positives or correct detections, calculated at an IoU threshold of 50%
- FN: false negatives or undetected objects, calculated at an IoU threshold of 50%

$$F1@50 = 2 * \frac{precision * recall}{precision + recall} \qquad [\text{IoU} \geq 50\%] \tag{S3}$$

$$AP@50 = \int_0^1 p(r)dr \qquad [\text{IoU} \geq 0.50] \tag{S4}$$

where:

- p: classwise detection precision for a given prediction confidence score, calculated at an IoU threshold of 50%
- r: classwise detection recall for a given prediction confidence score, calculated at an IoU threshold of 50%
- $p(r)$: classwise detection precision for a given detection recall score, calculated at an IoU threshold of 50%

$$mAP@50 = \frac{1}{n} * \sum_{i=1}^{i=n} AP_i@50 \tag{S5}$$

where:

- $AP_i@50$: AP@50 of class i
- n: number of detection classes

$$\text{mAP@50:95} = \frac{1}{10} * \sum_{t \; \epsilon \; \{0.50,\; 0.55,\; ...,\; 0.95\}} mAP@t \tag{S6}$$

where:

- $mAP@t$: mean average precision, calculated at an IoU threshold = t

5.2 Minimum required photography setup

$$\text{FoV}_{hor} = 2.\text{EWD}.tan(\frac{\text{AoV}_{hor}}{2}) \tag{S7}$$

where:

- FoV_{hor}: horizontal camera field of view [cm]
- EWD: effective working distance [cm]
- AoV_{hor}: horizontal camera angle of view [°]

$$\text{P} = \sqrt{\frac{FoV_{hor} * FoV_{ver}}{W_{sensor} * H_{sensor}}} * 10,000 \tag{S8}$$

where:

- P: pixel size [µm]
- FoV_{hor}: horizontal camera field of view [cm]
- FoV_{ver}: vertical camera field of view [cm]
- W_{sensor}: camera sensor width [pixels]
- H_{sensor}: camera sensor height [pixels]
- $W_{sensor} * H_{sensor}$: camera sensor quality [MP] $* 10^6$

$$EWD_{max} = \frac{P_{max}}{2 * 10,000} * \sqrt{\frac{W_{sensor} * H_{sensor}}{tan(\frac{AoV_{hor}}{2}) * tan(\frac{AoV_{ver}}{2})}} \tag{S9}$$

where:

- EWD_{max}: maximum effective working distance for species-level detection [cm]
- P_{max}: maximum pixel size for species-level detection [µm]
- W_{sensor}: camera sensor width [pixels]
- H_{sensor}: camera sensor height [pixels]
- $W_{sensor} * H_{sensor}$: camera sensor quality [MP] $* 10^6$
- AoV_{hor}: horizontal camera angle of view [°]
- AoV_{ver}: vertical camera angle of view [°]

$$AoV_{hor} = 2 * arctan(tan(\frac{AoV_{dia}}{2}) * \frac{W_{sensor}}{\sqrt{H_{sensor}^2 + W_{sensor}^2}}) \tag{S10}$$

where:

- AoV_{hor}: horizontal camera angle of view [°]
- AoV_{dia}: diagonal camera angle of view [°]
- W_{sensor}: camera sensor width [mm or pixels or relative]
- H_{sensor}: camera sensor height [mm or pixels or relative]

$$AoV_{ver} = 2 * arctan(tan(\frac{AoV_{dia}}{2}) * \frac{H_{sensor}}{\sqrt{H_{sensor}^2 + W_{sensor}^2}}) \tag{S11}$$

where:

- AoV_{ver}: vertical camera angle of view [°]
- AoV_{dia}: diagonal camera angle of view [°]
- W_{sensor}: camera sensor width [mm or pixels or relative]
- H_{sensor}: camera sensor height [mm or pixels or relative]

6 ADDITIONAL REFERENCES

Table S17. Additional references of the technical specifications and price estimations of the proposed photography setups.

sensor type + lens	reference
Raspberry Pi camera module 2	price: Amazon.com Inc., 2025<br>specs: Raspberry Pi Ltd., 2025
Raspberry Pi camera module 3	price: RaspberryStore, 2025<br>specs: Raspberry Pi Ltd., 2025
Arducam Pi Hawk-eye	price: Arducam, 2025<br>specs: Arducam Technology Co., Ltd., 2022<br>specs: Arducam, 2024
Samsung Galaxy A16 LTE<br>(main camera)	price: Samsung, 2025<br>specs: Samsung, 2025<br>specs: Camera FV-5, 2025
GoPro HERO13 Black + macro lens module<br>(16:9 - linear mode)	price: GoPro Inc, 2025<br>price: GoPro Inc, 2025<br>specs: GoPro Inc., 2025<br>specs: GoPro Inc, 2025<br>specs: GoPro Inc, 2025
iPhone 16 Pro<br>(ultra wide camera)	price: Apple Inc., 2025<br>specs: Apple Inc., 2025<br>specs: Apple Inc., 2025
Sony α7R III + FE 50mm F2.8 macro lens	price: Amazon.com Inc., 2025<br>price: Amazon.com Inc., 2025<br>specs: Sony Europe B.V., 2025<br>specs: Sony Europe B.V., 2025
